# Recent advances in bio-based production of top platform chemical, succinic acid: an alternative to conventional chemistry

**DOI:** 10.1186/s13068-024-02508-2

**Published:** 2024-05-29

**Authors:** Vinod Kumar, Pankaj Kumar, Sunil K. Maity, Deepti Agrawal, Vivek Narisetty, Samuel Jacob, Gopalakrishnan Kumar, Shashi Kant Bhatia, Dinesh Kumar, Vivekanand Vivekanand

**Affiliations:** 1 School of Water, Energy and Environment, Cranfield University, Cranfield, MK43 0AL UK; 2 Department of Bioscience and Bioengineering, Indian Institute of Technology Roorkee, Roorkee, Uttarakhand 247667 India; 3 Department of Chemical Engineering, School of Studies of Engineering and Technology, Guru Ghasidas Vishwavidyalaya (A Central University), Bilaspur, Chhattisgarh 495009 India; 4 Department of Chemical Engineering, Indian Institute of Technology Hyderabad, Kandi, Sangareddy, Hyderabad, Telangana 502284 India; 5 Biochemistry and Biotechnology Area, Material Resource Efficiency Division, CSIR-Indian Institute of Petroleum, Dehradun, Uttarakhand 248005 India; 6 Department of Biotechnology, School of Bioengineering, SRM Institute of Science and Technology, Kattankulathur, Chennai, Tamil Nadu 603203 India; 7 School of Civil and Environmental Engineering, Yonsei University, Seoul, 03722 Republic of Korea; 8 Department of Biological Engineering, College of Engineering, Konkuk University, Seoul, 05029 Republic of Korea; 9 School of Bioengineering & Food Technology, Shoolini University of Biotechnology and Management Sciences, Solan, Himachal Pradesh 173229 India; 10 Centre for Energy and Environment, Malaviya National Institute of Technology Jaipur, Jaipur, Rajasthan 302017 India

**Keywords:** Succinic acid, Bacteria, Yeast, Downstream processing, Chemo-catalysis, Commercial players, Circular bioeconomy

## Abstract

Succinic acid (SA) is one of the top platform chemicals with huge applications in diverse sectors. The presence of two carboxylic acid groups on the terminal carbon atoms makes SA a highly functional molecule that can be derivatized into a wide range of products. The biological route for SA production is a cleaner, greener, and promising technological option with huge potential to sequester the potent greenhouse gas, carbon dioxide. The recycling of renewable carbon of biomass (an indirect form of CO_2_), along with fixing CO_2_ in the form of SA, offers a carbon-negative SA manufacturing route to reduce atmospheric CO_2_ load. These attractive attributes compel a paradigm shift from fossil-based to microbial SA manufacturing, as evidenced by several commercial-scale bio-SA production in the last decade. The current review article scrutinizes the existing knowledge and covers SA production by the most efficient SA producers, including several bacteria and yeast strains. The review starts with the biochemistry of the major pathways accumulating SA as an end product. It discusses the SA production from a variety of pure and crude renewable sources by native as well as engineered strains with details of pathway/metabolic, evolutionary, and process engineering approaches for enhancing TYP (titer, yield, and productivity) metrics. The review is then extended to recent progress on separation technologies to recover SA from fermentation broth. Thereafter, SA derivatization opportunities via chemo-catalysis are discussed for various high-value products, which are only a few steps away. The last two sections are devoted to the current scenario of industrial production of bio-SA and associated challenges, along with the author's perspective.

## Introduction

**Fig. 1:**
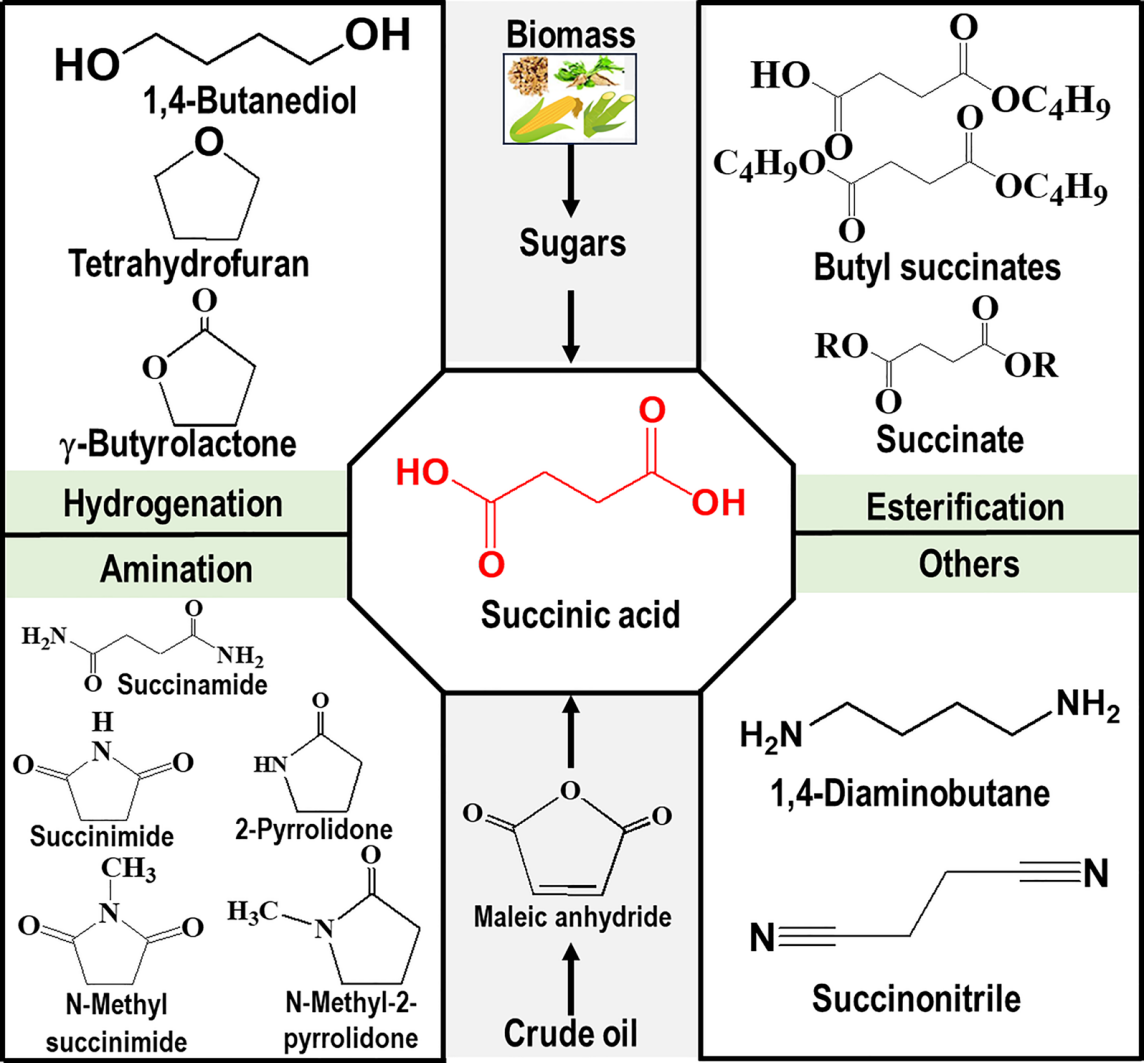
SA production and conversion to value-added derivatives

Since the inception of crude oil, the chemical sector has been tightly interwoven with the fossil industry as the latter caters both the feedstock and energy demand for manufacturing all types of organic chemicals. Despite robust market demand, the chemical industry is unsustainable owing to heavy reliance on non-renewable raw materials and is the third largest CO_2_ emitter, thereby imposing significant negative environmental impact. Hence, there is a compelling need for the transition toward clean and green biochemicals with low-carbon emissions, and it is anticipated that the share of bio-based products, especially building block chemicals, will continuously grow in the near future. The US Department of Energy (DoE) prepared a list of top platform chemicals that are obtainable from biomass. The original list was published in 2004 and was revised in 2009. Succinic acid (SA), a dicarboxylic acid (C_4_H_6_O_4;_ MW: 118.09 g/mol), was present in the original list and retained its position in the revised list as well, showing the commercial significance of this organic acid [1–3]. The presence of two carboxyl groups on the terminal carbon atoms confers versatile derivatization functionality to SA with vast applications in food and beverages (acidulant, flavorant, and sweetener), polymers (polybutylene succinate, polybutylene succinate–terephthalate, and polyester polyols), paints, and pharmaceutical industries [4–6]. The SA can be transformed into several valuable chemical compounds, such as 1,4-butanediol, succinimide, succinonitrile, tetrahydrofuran (THF), 2-pyrrolidone, etc., using chemo-catalysis (Fig. 1). SA and its derivatives find myriad applications in the production of green solvents, surfactants, detergents, lacquer, perfumes, fragrances, coolants, synthetic resins, pigments, biodegradable polymers, and plasticizers [5, 7–9]. The global market of bio-based SA is expanding rapidly, and during the forecast period between 2017 and 2030, it is predicted to hit US $ 900 million with a compound annual growth rate of 19.6 % [10].

The petrochemical SA production routes include catalytic hydrogenation or electrolytic reduction of maleic acid or maleic anhydride, which are obtained by oxidation of benzene or butane, resulting in succinic anhydride, which on hydrolysis forms SA [4, 11]. However, these routes use non-renewable feedstocks and expensive metal-based catalysts, require high temperature and pressure, and suffer from low yield and inferior product quality. All these factors, including concerns related to global climate change owing to high greenhouse gas (GHG) emissions, have triggered the search for alternative sustainable and environmentally friendly pathways for SA synthesis [11, 12]. SA is an intermediate of the TCA/Glyoxylate cycle, which is prevalent in biological systems, including plants, humans, and microorganisms. Biological SA production is a cleaner, greener, and promising alternative to petrochemical technology [13]. In this regard, the last decade witnessed the commercialization of bio-based SA production wherein several industrially potent microbial systems were evaluated using renewable carbonaceous feedstocks.

Biomass-derived SA is associated with low carbon footprints fueled by CO_2_ sequestration, as commonly used biological routes for SA synthesis require CO_2_ as a co-substrate. Recycling renewable carbon of biomass (an indirect form of CO_2_) along with capturing atmospheric CO_2_ offers carbon-negative SA biomanufacturing technology to reduce atmospheric CO_2_ load [4, 5, 9]. For example, the carbon emission from fossil-based SA production is 1.94 kg CO_2_ eq./kg SA, while it is merely 0.88 kg CO_2_ eq./kg SA when produced from glucose via microbial route [6, 14]. Therefore, bio-based SA manufacturing can reduce > 60% of GHG emissions compared to fossil-based SA production [11, 15]. However, COVID-19 pandemic exerted an adverse effect on the bio-based SA market in 2020 [16]. Despite the high potential of bio-based SA production, the market analysis indicates that the high price of bio-SA (US $ 2.94/kg) compared to fossil-derived SA (US $ 2.5/kg) limits its future market developments [17–19]. These techno-commercial issues can be overcome by using a large variety of abundant and cheaper natural bioresources, making biological SA cost-competitive with fossil-based SA [19, 20].

The review begins with the biochemistry of the major pathways involved in accumulating SA as an end product. It comprehensively discusses the most recent global research efforts to attain high titer of bio-based SA using native and engineered bacterial and yeast strains. The review outlines the details of pathway/metabolic, evolutionary, and process engineering approaches adopted for enhancing the TYP (titer, yield, and productivity) metrics of SA. Thereafter, it discusses the advantages and limitations of conventional downstream processing (DSP) strategies, citing newly developed processes for SA recovery as the final product. The versatility of SA as a platform chemical is later demonstrated by showcasing the chemo-catalytic upgradation of SA to several valuable products, with emphasis on different heterogeneous catalysts, reaction mechanisms, and product yield/selectivity under different reaction environments. The last two sections are devoted to the current scenario of industrial production of bio-SA and associated challenges, along with the author's perspectives.

## Biochemistry and physiology of SA production

**Fig. 2:**
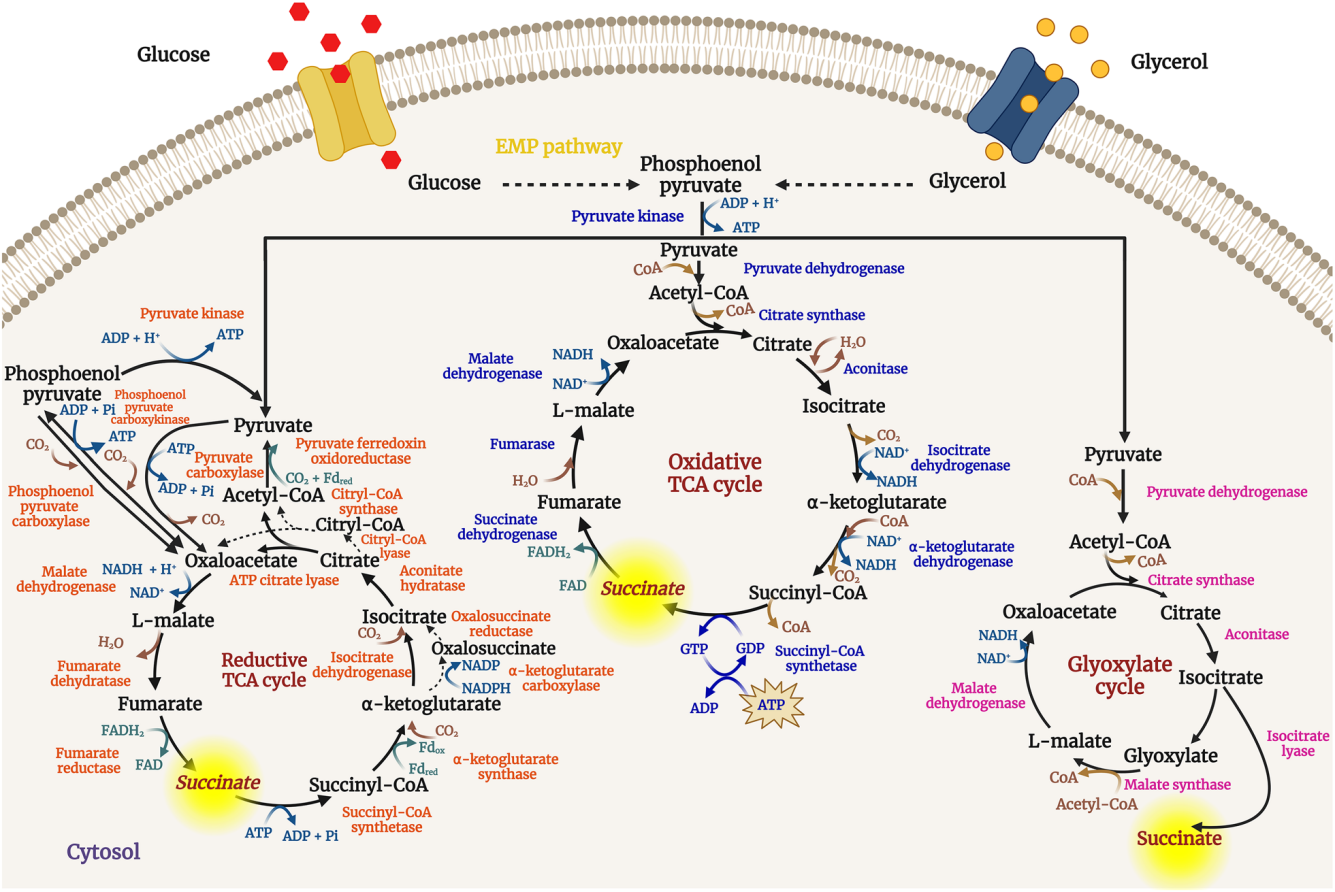
Different metabolic pathways of microbial SA production [23, 24]

Bio-based SA is produced by microbial degradation (aerobic and/or anaerobic consumption) of hexose and pentose sugars as carbon sources [4, 6]. On the basis of the degree of reduction, the maximum theoretical yield of SA for glucose, xylose, and glycerol is 1.71, 1.43, and 1.0 mol/mol, respectively, depending on electron availability. For example, the theoretical yield can be enhanced to 2.00 mol in the case of glucose, if CO_2_ and additional reducing power are supplied [4, 21, 22]. The three metabolic pathways for SA biosynthesis include oxidative TCA, reductive TCA (rTCA), and glyoxylate cycle (Fig. 2).

*Oxidative TCA cycle:* SA is an intermediate of the oxidative TCA cycle, and the physiological role of the pathway is to completely oxidize two carbon atoms unit acetyl-CoA into CO_2_. The two main products of the cycle are carbon dioxide and reducing equivalents (NADH/FADH_2_). Therefore, the cycle is primarily active under aerobic conditions to oxidize NADH/FADH_2_ back into NAD^+^/FAD. The cycle starts with condensation of acetyl-CoA with oxaloacetate (OAA) to give citrate, which is isomerized to isocitrate (Fig. 2). The next two steps involve oxidative decarboxylation of isocitrate to α-ketoglutarate and α-ketoglutarate to succinyl-CoA. Furthermore, succinyl-CoA is converted into SA with production of GTP, and the reaction is catalyzed by succinyl-CoA synthetase. In the next step, SA is further dehydrogenated to fumaric acid with mediation through succinate dehydrogenase (Fig. 2), and this step should be blocked or inactivated to accumulate SA in high amounts via the oxidative TCA cycle [6, 25]:

Succinyl-CoA + GDP + Pi → SA + GTP + CoA-SH; Succinyl-CoA synthetase.

SA + FAD → Fumaric acid + FADH_2_; Succinate dehydrogenase.

The overall equation for SA production from oxidative TCA cycle is as follows:

Acetyl-CoA + OAA + 2NAD^+^  + GDP + Pi → SA + 2NADH + 2H^+^  + 2CO_2_ + CoA-SH + GTP.

Since the reaction generates two moles of NADH for every mole of SA produced, continuous aeration is required to regenerate NAD^+^ for smooth production of SA via this route.

*Reductive TCA cycle: * It is also known as the reverse rTCA cycle. It is one of the six autotrophic pathways found in nature and predominates under anaerobic conditions, where SA acts as the terminal electron acceptor. The pathway starts with the carboxylation of pyruvate or phosphoenol pyruvate (PEP) to OAA. The commonly used enzymes for the carboxylation of C3 metabolites are PEP carboxykinase (PEPCK), PEP carboxylase (PEPC), and/or pyruvate carboxylase (PYC); however, PEPCK is considered to be the key enzyme for the production of SA with subsequent ATP formation [4, 26]. OAA is reduced to malate [malate dehydrogenase (*mdh*)], followed by dehydration of malate to fumarate [fumarase (*fr*)], which is further reduced to SA by fumarate reductase [fumarate reductase (*frd*)] (Fig. 2). PEPC/PEPCK/PYC replenishes OAA through CO_2_ fixation, and this step of carboxylation is an advantageous feature of the pathway as it enables the capturing of carbon dioxide, a primary GHG. Enhanced CO_2_ levels improve SA production by diverting more carbon flux towards SA via OAA. Contrary to the oxidative pathway, the reductive route consumes NADH and requires a surplus of NADH in the case of traditional carbohydrates, where SA production is not redox balanced. Theoretically, two moles of SA can be obtained from one mole of glucose and synthesis of each mole of SA requires two moles of NADH, but one mole of glucose produces only two NADH moles. This shortage of two extra moles of NADH impedes higher SA accumulation and suggests that additional reducing power should come from other parts of metabolism [4, 25]. However, the pathway is balanced in the case of glycerol which is a more reduced carbon source:

Glucose + 2NAD^+^  → 2Pyruvate/PEP + 2NADH + 2H^+^

Pyruvate/PEP + CO_2_ + 2NADH + 2H^+^ → SA + 2NAD^+^  + H_2_O.

The sequence similarity exists between *frd* and succinate dehydrogenase (*sdh*) of oxidative TCA cycle. They are similar in composition and subunit structure and catalyze the interconversion of fumarate to succinate in opposite directions. The enzyme characterization also revealed that the functional characteristics, substrate specificity, and enzyme kinetics were similar between *frd* and *sdh* enzymes.

The oxidative and reductive TCA cycles differ in terms of thermodynamic feasibility, maximum theoretical yield, and role of CO_2_. The maximum theoretical yield, which could be achieved via the reductive TCA cycle, is 1.71, while in the case of the oxidative TCA cycle, it is 1.0 mol SA/mol glucose. The oxidative TCA cycle is thermodynamically feasible; in contrast, the reductive branch is non-feasible [26, 27]. The third and most important difference is that reductive pathways require CO_2_ as a co-substrate as SA is formed through carboxylation of C3 metabolites (PEP and/or pyruvate). On the other hand, in the case of oxidative route CO_2_ is released as a byproduct. One mole of CO_2_ is consumed for every mole of SA synthesized in reductive pathways, while the oxidative cycle releases two moles of CO_2_ for the production of one mole of SA [4, 6].

*Glyoxylate cycle:* It is an anabolic pathway occurring in plants, bacteria, protists, and fungi. The glyoxylate cycle is a modification of the tricarboxylic acid and bypasses the decarboxylation steps where CO_2_ is lost. The cycle enables the microorganisms to grow on  C2 carbon sources to avoid carbon loss when traditional carbon sources such as glucose are not available. The two key enzymes of the cycle are isocitrate lyase and malate synthase. Isocitrate is cleaved into SA and glyoxylate, which combines with another acetyl-CoA to provide malate (Fig. 2). Each turn of the cycle consumes two moles of acetyl-CoA and generates one mole of SA [6, 25]:

Acetyl-CoA + OAA → Isocitrate + CoA-SH.

Isocitrate → SA + Glyoxylate (Isocitrate lyase).

Glyoxylate + Acetyl-CoA → Malate + CoA-SH (Malate synthase).

Malate + NAD^+^  → OAA + NADH.

*Overall reaction* 2 Acetyl-CoA + NAD^+^  → SA + 2CoA-SH + NADH.

The glyoxylate cycle results in the net generation of NADH and is essentially active under aerobic conditions where NADH can be recycled to NAD^+^. The use of the glyoxylate cycle for SA biosynthesis results in higher yields than the oxidative TCA pathway as decarboxylation steps are bypassed. Furthermore, the glyoxylate cycle is located outside the mitochondria, and it eliminates the problems associated with the mitochondrial transport of SA [28, 29].

## Native producers of SA

State of the art studies reveal that primarily prokaryotes belonging to diverse genera such as *Actinobacillus*, *Anaerobiospirillum*, *Basfia*, *Corynebacterium, Manheimia*, *Enterobacter*, *Bacillus*, and *Enterococcus are* natural hosts for SA production. All these native producers accumulate SA as the end product and many of them have been isolated from the cattle rumen. SA acts as an important precursor for the biosynthesis of propionic acid, which constitutes 20% of total volatile fatty acids (VFAs). The propionic acid generated is absorbed by the rumen wall and further oxidized for milk production and energy generation [11, 30]. Though SA is an intermediate of the TCA cycle, native producers have the capability to produce SA as a fermentative end product from the anaerobic metabolizing *A. succiniciproducens*, *A. succinogenes*, *M. succiniciproducens,* etc. Tables 1 and 2 summarize SA production by native and non-native engineered bacterial and yeast strains, respectively. These anaerobic SA-accumulating microorganisms use a reductive TCA cycle for manufacturing SA, and work best near the neutral pH [4, 30, 31]. They have the ability to fix CO_2_, and many of them are capable of fumarate respiration as well. Ubiquinone and menaquinone are electron carriers used for aerobic and anaerobic respiration, respectively. Most of these native producers, including *A. succinogenes* and *M. succiniciproducens,* contain pathways for menaquinone but not for ubiquinone. Menaquinone uses fumarate as an electron acceptor, generating SA as a product of the reaction catalysed by fumarate reductase [32, 33]. PEP is the important branching point in natural producer and the key enzyme involved in SA production is PEPCK (encoded by *pckA* gene), a powerful CO_2_ fixing enzyme that catalyses the conversion of PEP to OAA while producing ATP (PEP + CO_2_ + ADP → OAA + ADP). SA production via the reductive pathway is strongly influenced by CO_2_ levels, and the CO_2 -_rich environment of the bovine rumen facilitates SA production [34]. In the succeeding paragraphs, the native SA producers and the recent progress made to exploit their metabolic potential has been discussed in an elaborative manner:

**Table 1:** Recent state of the art involving native succinic acid producers that produced ≥ 50 g/L succinic acid

Microorganism	Genotype	Feedstock	Fermentation mode	Succinic acid			Reference
				Titer (g/L)	Yield (g/g)	Productivity (g/L. h)	
* A. succinogenes*	Wild type	Glucose	Fed-batch	100.5	1.00	1.70	[35]
		Cassava roots	Fed-batch	151.4	1.51	3.22	
		Duckweed hydrolysate	Batch	75.5	0.83	1.35	[36]
		Molasses	Fed-batch	83.7	0.93	1.74	[37]
		Glucose	Repeated-batch (immobilized cells)	107.0	0.73	1.49	[38]
	*↑pepc*	Glucose	Batch	59.47	0.79	0.99	[39]
	*∆Asuc_914*	Glucose	Fed-batch	71.92	1.03	1.20	[40]
*A. succiniciproducens*	Wild type	Glucose	Continuous	83.0	0.89	10.4	[41]
*B. succiniciproducens*	*∆pfl*	Glycerol + Maltose	Batch	64.7	–	2.69	[42]
	*ΔfruA*	Sucrose	Fed-batch	71.0	0.74	–	[43]
*M. succiniciproducens*	*∆ack-pta ∆pfl ∆ldh ↑fdh*	Glucose + FA	Fed-batch	69.8	0.93	2.79	[31]
		Glucose + Glycerol + FA	Fed-batch	72.0	1.03	2.88	
		Sucrose + FA	Fed-batch	76.1	0.84	4.08	
	*∆ldhA ∆pta ∆ackA ↑mdh*	Glucose + Glycerol	Fed-batch	101.2	0.90	4.18	[33]
		Glucose + Glycerol	Fed-batch	134.3	0.82	10.3	
	*ΔldhA Δpta ΔackA*↑*pelB* ↑*cti(pae)*	Glucose	Fed-batch	84.21	1.27 (molar)	3.20	[44]
		Glucose + Glycerol	Fed-batch	97.1	1.26 (molar)	3.01	
*C. glutamicum*	*ΔldhA ΔackA-pta Δpqo Δcat ↑Psod-ppc ↑Psod-pyc ↑xylA ↑xylB ↑tal ↑tkt ↑araE ↑pyc ↑gltA ↑sucE*	Glucose and Xylose	Batch	100.2	0.82	4.36	[45]
*C. glutamicum*		Corn stover hydrolysate (glucose + xylose)	Batch	98.6	0.98	4.29	
*C. glutamicum*	**↑***pyc ΔldhA*	Glucose	Fed-batch	146.0	0.92	3.17	[46]
*C. glutamicum*	Wild type	Glucose	Fed-batch	93.6	0.60	1.42	[47]
*C. glutamicum*	*Δldh ΔpoxB Δpta-ackA ΔactA Δpck ΔptsG *↑*pyc*^P458 ^↑P_tuf_::*pckG *↑P_tuf_::*ppc *↑*NCgl0275*	Glucose	Fed-batch	152.2	1.10	1.11	[48]
*C. glutamicum*	*ΔptsG ΔiolR ↑ioT1 ↑ppgk*	Glucose	Fed-batch	90.8	0.97	1.89	[49]
*C. glutamicum*	*Δldh Δpta-ackA Δcat ↑pyc ↑ppc ↑Ncgl0275*	Glucose	Fed-batch	117.8	0.59	1.04	[50]
	*Δldh Δpta-ackA Δcat ↑pyc ↑ppc ↑Ncgl0275 ↑xylBc ↑xylXc ↑xylCc ↑xylDc ↑xylAc ↑xylA ↑xylB ↑xylE*	Glucose and Xylose	Batch	64.2	0.69	1.17	
		Corn stover hydrolysate (glucose + xylose)	Batch	64.2	0.76	1.07	
*C. glutamicum*	*Δcat ΔpqoΔpta-ackA ΔldhA ↑pyc*	Glucose	Pulse-feed (oxygen limited conditions)	78	1.37 (molar)	1.08	[51]
*E. aerogenes* LU2	Wild	Lactose	Batch	51.35	0.53	0.35	[52]
		Whey permeate		57.7	0.62	0.34	
Strain AKR177	Wild	Pure Glycerol	Batch	117	1.3	0.34	[76]
		Crude Glycerol		86.9	0.9	0.33	
*B. velezensis*	Wild	Glucose	Batch	50.2	0.936	1.04	[75 ]
* E. gallinarum*	Wild	Glucose		66.9	1.12	1.11	
	Wild	Palm oil mill waste water + Molasses (80:20)		73.9	3.87	1.23

**Table 2:** State of the art showing succinic acid production from non-native bacterial and yeast strains

Microbes	Genotype	Feedstock	Fermentation mode	Succinic acid			Reference
				Titer (g/L)	Yield (g/g)	Productivity (g/L. h)	
Engineered bacterial strains							
* E. coli*	*ΔpflA Δldh ΔptsG *↑*pyc*	Glucose	Fed-batch	99.2	1.1	1.30	[53]
* E. coli*	*∆ldhA ∆adhE ∆ackA ∆focA ∆pflB*	Glucose	Batch	86.6	0.93	0.90	[54]
* E. coli*	*∆ldhA ∆pflB ∆focA Δpta-ackA ↑pck ↑ptxD*	Glucose	Two-stage fermentation	137	1.0	1.43	[55]
* E. coli*	*ΔldhA ΔpflB ↑pck*	Crude glycerol pretreated with activated charcoal	Two stage fermentation	66.78		0.70	[56]
		Pure glycerol		72.67		0.71	
* E. coli*	*∆ldhA ∆pflB ∆pts ∆glpK ∆dhaKLM *↑*galP *↑*pck *↑*dhaK*	Glycerol	Batch	57	1.18	0.59	[57]
* E. coli*	*∆ldhA ∆pflB ∆ppc ∆ptsG *↑*pepck*	SCB hydrolysate (glucose + xylose)	Repetitive batch	83.0	0.87	2.31	[58]
* E. coli* FZ661T	*ΔldhA ΔadhE ΔiclR ΔackA-pta Δptsg ΔlacI ↑pycA ↑fdh1 (galR replaced by pTrc‐galP)*	Glucose:Galactose: Fructose (1:1:1)	Fed -batch	95.8	–	1.74	[59]
		Galactose fortified soybean molasses hydrolysate		74	1.15	1.6	
		Glucose + xylose	Fed-batch	107		1.74	[60]
		Wood hydrolysate	Batch	54.5		1.81	
* E. coli* KJ122	*∆ldhA ∆adhE ∆ackA ∆focA ∆pflB ∆mgsA ∆poxB ∆tdcDE ∆citF ∆aspC ∆sfcA*	NaOH pretreated rice straw	Batch SSF	69.8	0.84	0.78	[61]
			Fed-batch SSF	103.1	0.87	1.37	
		Dried cassava pulp	Batch SSF	80.86	0.70	0.84	[62]
			Fed-batch SSF	98.63	0.71	1.03	
* K. oxytoca* KC004- T160	*ΔadhE Δpta-ackA ΔldhA ΔbudAB ΔpflB* followed by adaptive evolution	Glucose		82.88	0.83	0.58	[63]
		Sugarcane molasses		57.5	0.84	0.48	
* V. natriegens*	*Δlldh Δdldh Δpfl Δald* *Δdns::pycCg*	Glucose	Anaerobic zero growth fermentation	60.4	1.14 (molar)	8.6	[64]
Engineered yeast strains							
* Y. lipolytica*	*∆SDH5*	Crude glycerol	Fed-batch	160.2	0.40	0.40	[65]
* Y. lipolytica*	*∆SDH5 ∆ach* ↑*PEPCK* ↑*SCS2*	Glycerol	Fed-batch	110.7	0.53	0.80	[66]
* Y. lipolytica* PSA02004	↑*Ylsdh5* followed by adaptive evolution via cell immobilization	Glucose	Batch	65.7	0.50	0.69	[67]
		Food waste hydrolysate		87.9	0.56	0.70	
* Y. lipolytica* PGC62‑SYF‑Mae	∆*SDH5* ∆*ach* ↑*PEPCK* ↑*SCS2 *↑*TbFrd *↑*YlScs2 *↑*YlYhm2-YlMls-YlIcl *↑*SpMae1*	Glucose	Fed-batch	101.4	0.37	0.70	[68]
* S. cerevisiae*	**↑***PYC2*** ↑***MDH3R* **↑***FumC***↑***FRDS1 ∆fum1 ∆gpd1 ∆pdc*	Glucose	Batch	13.0	0.14	0.11	[69]
* S. cerevisiae*	*∆GUT1*** ↑*** GDH***↑***DAK1*** ↑***MDH3-R ***↑***fumR ***↑***FRDg-R ***↑**DCT-02	Glycerol	Batch	10.7	0.22	0.064	[70]
* S. cerevisiae*	*∆GUT1*** ↑*** GDH***↑***DAK1*** ↑***MDH3-R ***↑***fumR ***↑***FRDg-R ***↑***PYC2* **↑**DCT-02	Glycerol	Batch	35.0	0.60	0.36	[71]
* S. cerevisiae*	*∆SDH1 ∆SDH2 ∆IDH1 ∆IPH2*	Glucose	Batch	3.62	0.11	0.022	[72]
* I. orientalis*	*↑pyc↑mdh↑fumr↑frd↑SpMAE∆pdc∆gpd ∆g3473↑PaGDH↑DAK∆g3837*	Sugarcane juice medium	Fed-batch	104.6	0.63	1.25	[73]
		Glucose + Glycerol		109.5	0.65	0.54	

*Actinobacillus succinogenes*: *A. succinogenes* is a capnophilic ruminal facultative anaerobe, non-pathogenic, and gram-negative bacterium. *A. succinogenes* is the most promising and versatile biofactory employed for SA production. The bacterium is classified as a biosafety level 1 microorganism and considered as an industrially potent microbial strain for SA production [4, 5, 26]. *A. succinogenes* can metabolize a variety of carbon sources, such as glucose, xylose, arabinose, mannose, galactose, fructose, sucrose, lactose, cellobiose, mannitol, maltose, and glycerol. This keeps *A. succinogenes* in a superior position as most of these carbon sources are abundant in crude renewable sources. Furthermore, the bacterium exhibits good tolerance to fermentation inhibitors, making it a promising biocatalyst for integrated biorefineries. Glucose is catabolized to PEP/pyruvate by Glycolytic and oxidative pentose phosphate pathway in *A. succinogenes*. The bacterium has a partial TCA cycle and lacks key enzymes, such as citrate synthase, isocitrate dehydrogenase, and α-ketoglutarate dehydrogenase, impeding SA production through oxidative cycle. Glyoxylate cycle is absent in the fermentative metabolism of *A. succinogenes,* and the active pathway leading to SA accumulation is the reductive branch of the TCA cycle, with PEPC as the key enzyme connecting C3 and C4 pathways. Acetate, ethanol, and formate coming from the metabolism of PEP/pyruvate are obtained as main byproducts during SA production [4, 9].

Here, we are going to discuss a few important reports, wherein industrial SA titers were obtained from *A. succinogenes.* Either the researchers experimented using low cost renewable feedstocks or employed strategies like bioprocess intensification, cell immobilization, using electrical current and strain modification. For instance, Thuy et al. investigated SA bioproduction of SA by *A. succinogenes* using cassava root, a starchy feedstock that is low-hanging fruit in terms of fermentable sugars [35]. Besides sugars, it also contains other valuable nutrients, such as proteins, vitamins, and minerals. The saccharification of cassava roots was performed using commercial enzymes, including Liquozyme, Spirizyme, and Viscozyme. The SA accumulated during batch culture using cassava root was 93.3 g/L with a conversion yield of 0.77 g/g and productivity of 1.87 g/L. h, while numbers achieved with pure glucose were 73.0 g/L, 0.60 g/g, and 1.46 g/L. h. In the case of fed-batch culture, TYP metrics with cassava root and pure glucose were 151.4 g/L, 1.51 g/g and 3.22 g/L. h and 100.5 g/L, 1.0 g/g and 1.70 g/L. h, respectively. This massive SA titer amassed is the highest reported till date for bio-SA production. The upstream work was followed by DSP, where impurities (proteins, macromolecule, and multivalent ions) were removed by nanofiltration (NF). The SA was recovered from a mixture of organic acids using seeded batch cooling crystallization. The SA recovered had a crystal purity and crystallinity of 99.4% and 96.8%, respectively.

Like cassava roots, duckweed is yet another promising second-generation (2G) feedstock, which does not compete with edible food and arable land and is rich in starch [74]. However, the recent study carried out by Shen et al. confirms the presence of other non-starchy polysaccharides (cellulose, pectin etc.) in duckweed, which results in a highly viscous solution, creating mass transfer problems and incurring high power consumption [36]. To overcome this issue, they employed SSSF (semi-simultaneous saccharification and fermentation), which includes a short saccharification period using combined enzyme mixture (Viscozyme L and Pectinex Ultra SP-L) with a high hydrolytic rate before SSF starts [36]. The enzymatic pretreatment, fermentation configuration, and initial substrate concentration were optimized. The SSSF outperformed SHF and SSF in terms of SA production. The batch bioreactor fermentation with SSSF mode and initial substrate concentration of 180 g/L yielded 75.5 g/L SA in 56 h. The SA yield was 0.42 g/g duckweed, with productivity of 1.35 g/L. h [36].

SA production is often constrained by the availability of reducing power, and one of the methods to alleviate this is the use of microbial electrolysis cells (MEC), where hydrogen is produced from oxidation of organic compounds by microorganisms. These MEC are known to improve SA yield by enhancing the levels of intracellular NADH [75]. Wang et al. made use of MEC for SA production using molasses as carbon source, and different pretreatment methods were used to remove suspended impurities and heavy metals present in molasses [37]. Among them, the anionic polyacrylamide method prevented the accumulation of metal ions at the cathode, thereby facilitating biomass and SA formation, was found to be the best one. MECs need an input potential and constant voltage to generate electrons to elevate sufficiently high NADH levels. They achieved a SA titer of 83.7 g/L through fed-batch fermentation using molasses as feedstock in MEC at a constant voltage of − 1.0 V with corresponding yield and productivity of 0.93 g/g and 1.74 g/L. h, respectively.

It is highly desirable that the use of microbial cells acting as catalysts could be prolonged. In yet another study, Corona—González et al. immobilized *A. succinogenes* on agar beads, enduring gentle shaking and continuous diffusion of CO_2_ during SA production from glucose [38]. The longevity of performance was assessed through repeated batch fermentation using immobilized cells on agar beads. After five fermentation cycles with a total time of 72 h, 147.6 g/L of glucose was metabolized, leading to a SA concentration of 107 g/L. In the most recent study, Chen and Zheng altered several genes in *Actinobacillus* strain which were anticipated to play a crucial role in the microbial growth and SA production using pLGZ922 expression vector and a cytosine base editor (CBE) based on CRISPR/Cas9 [39].Their study revealed that when two of the genes, namely, *pyc* (pyruvate carboxylase) and *pepc* (phosphoenolpyruvate carboxylase) from *Corynebacterium acetoacidophilum*, which were instrumental in CO_2_ fixation were individually over-expressed in *A. succinogenes*, the SA titers increased from 52.35 to 55.66 and 59.47 g/L, respectively. Despite a delayed growth, the SA yields were enhanced from 0.70 g/g to 0.82 and 0.79 g/g, respectively. Furthermore, altering the pathways of acetate, formate and deletion of OAA decarboxylase had no impact of SA biosynthesis. It is the first study wherein data mining of certain sugar and SA transporters was done. When two genes that encoded for two SA exporters, namely, Asuc_0716 and Asuc_0715, were individually knocked out, it had a prominent and deleterious effect on both cell homeostasis and SA biosynthesis [39]. In the same year Chen et al., developed an efficient, fast and precise gene manipulation toolkit for editing the genes of *Actinobacillus* by developing series of specific base editors (BE’s) by fusing Cas nuclease and cytidine/adenine deaminase [40]. When they used BE’s to delete the gene encoding of glucose transport (*Asuc_0914*), which shared homology with *ptsG* gene (encoding glucose permease) in *E. coli*, they found a 1.24 fold increase in titer and yield of SA compared to parent strain. In a 3L bioreactor, the ΔAsuc_0914 strain accumulated a maximum of 71.92 g/L SA with yield and productivity being 1.03 g/g and 1.18 g/L/h, respectively [40].

These kinds of fundamental studies have opened new avenues to implement metabolic engineering strategies using CRISPR–Cas system wherein little is known about the genetic makeup of organism and it displays weak DNA repair ability besides lack of expression plasmids and promoters for expressing guided RNA. Both the studies have further helped in identifying the critical role of transporters in *Actinobacillus* and opened new avenues for gene editing which were not exploited optimally till date for enhanced SA production.

*Anaerobiospirillum succiniciproducens:* *A. succiniciproducens* is an obligate anaerobe with the ability to produce SA in high yield and reduced byproduct formation. The bacterium can use a wide spectrum of carbohydrates as a carbon and energy source and ferment them into mixed acids (succinic, acetic acid, and lactic acid) and ethanol [76–78]. The commercial potential of this strain was realized way back in 1996 when Michigan Biotechnology Institute obtained a US patent, wherein fluoroacetate-resistant variants of *A. succiniproducens* were developed that accumulated high SA titers and produced acetic acid in low concentrations, a byproduct of the bioprocess. It was claimed in the invention that one of its potential variants, FA-10, was capable of producing 55 g/L of SA with high productivity when grown on dextrose [79]. Despite this initial breakthrough, the literature on SA production by *A. succiniciproducens* is scarce. There is only one isolated report in recent times wherein Meynial-Salles et al. designed a three-stage continuous process for SA production by integrating membrane cell recycling and electrodialysis system with a bioreactor to attain industrial SA titers [41]. The anaerobic fermentations suffer from low biomass concentration and a continuous process equipped with recycling of cell factories or biocatalysts is a powerful method to enhance volumetric productivity. Furthermore, to alleviate product inhibition, a mono-polar electrodialysis pilot was integrated with the cell recycle bioreactor, which allowed the recycling of organic acid-depleted permeate after the removal of acetic acid and SA and abolished the growth inhibition phenomenon caused by organic acid toxicity. This integrated system runs at a dilution and cell bleeding rate of 0.93 h^−1^ and 0.03 h^−1^, respectively, resulting in a concentrated solution containing 83 g/L SA and 19 g/L acetate. The conversion yield and productivity were 0.89 g/g and 10.4 g/L. h, respectively.

*Basfia succiniciproducens:* It is a non-pathogenic, gram-negative, facultative anaerobic, and capnophilic bacterium belonging to the *Pasteurellaceae* family [80]. Like *Mannheimia succiniciproducens*, *B. succiniciproducens* makes use of the oxidative and reductive branches of the TCA cycle to generate SA, which is different to well-characterized *A. succinogenes* accumulating SA via the reductive branch of the TCA cycle [32]. Furthermore, it can assimilate a variety of carbon sources, such as glycerol, sucrose, glucose, fructose, xylose, arabinose, galactose, and mannose [81]. The byproducts generated during SA production are lactic, formic and acetic acid. Like *A. succiniciproducens*, *B. succiniciproducens* is an extremely attractive biofactory for accumulating SA. Despite an attractive host among native producers for SA production, the literature on *B. succiniciproducens* is quite scarce and in fact, several reviews on biological SA production do not discuss about it. Therefore, more work is required to decode the potential of this microbe [81].

In the past one decade, BASF has been granted on number of patents where they have developed a number of genetically modified strains of *B. succiniciproducens* that can thrive on variety of carbon substrates. One of their most recent and notable work is a US patent on SA producing *B. succiniciproducens* in which the *pfl* gene of the wild strain (DD1) was knocked out. This recombinant strain (LU15348) grew well on glycerol and, with maltose as co-substrate, could accumulate a maximum of 64.7 g/L SA with productivity being 2.69 g/L/h. The concentrations of acetic acid, formic acid, pyruvic, and malic acid were < 1 g/L, and lactic acid was the only predominant byproduct with concentration being 2.5 g/L. The invention further discloses the DSP of SA, where they could recover its crystals with 99.8% purity. Thus, BASF has demonstrated the industrial feasibility of the strain for SA production [42]. Earlier, the same wild strain DD1 was genetically manipulated for the production of sucrose-based bio-SA. Using powerful tool like ^13^C metabolic flux analysis, a precise and workable strategy was devised, *fruc*A gene encoding for fructose phosphotransferase system (PTS) was deleted, which was a phosphoenolpyruvate (PEP) dependent enzyme that diverted PEP away from SA formation. When the tailored recombinant strain was tested with sucrose as the sole carbon source under fed-batch conditions, it produced 71 g/L of SA, with only 7.3 g/L lactic acid as byproduct [43].

There are a few reports wherein crude lignocellulosic hydrolysates have been attempted for SA production from *B. succiniciproducens,* but titers attained were relatively lower compared to pure carbon feedstocks*.* For example, in the year 2016 Salvachúa et al. investigated the prospects of *B. succiniciproducens* to accumulate SA on glucose, xylose, mock sugars (glucose, galactose, xylose, and arabinose), mock and real xylose-rich DDPAH (deacetylated dilute acid pretreated hydrolysate) (glucose, galactose, xylose, arabinose, acetate, furfural, and HMF) from corn stover at different levels (40, 60, 80, and 100 g/L) [82]. SA titer obtained on most of these sugars was in the range of 25–31 g/L with acetic, formic, and lactic acid as major byproducts. In the case of real DDPAH (60 g/L), there was a lag phase of ~ 24 h, but once hydrolysate was biologically detoxified, the SA titers peaked at 30 g/L, which productivity being 0.42 g/L/h. The study revealed that like *A. succinogenes*, *B. succiniciproducens* also has the ability to detoxify furan derivatives by reducing them to their corresponding alcohols [82].

Likewise, in the year 2019 Cimini et al. [83], evaluated the process efficiency of a fed-batch fermentation with at a pilot scale (150 L) where *B. succiniciproducens* BPP7 was grown on *Arundo donax* hydrolysate containing glucose (28.9 g/L), xylose (15.6 g/L) and acetic acid (5.6 g/L). The highest SA titer of 37 g/L was achieved when *A. donax* hydrolysate (14.5 g/L glucose + 8.5 g/L xylose) mixed with 19.5 g/L pure glucose was added at 0.8 g/L.h. The fermentation lasted for 43 h with an overall yield being 0.9 g/g. The material flow analysis revealed that combined efficiency of pretreatment and hydrolysis of *A. donax* was 54% and finally 88.5% SA was obtained/kg used biomass but overall output being 52% [83]. In the year 2023, *B*. *succiniciproducens* ATCC 22022 was evaluated to valorize glucose-rich enzymatic hydrolysate derived from sulphite derived sludge, a side waste product of paper and pulp industry. Under batch mode, the strain was able to produce 30.6 g/L SA with yield and productivity being 0.52 g/g and 0.63 g/L/h, respectively, when hydrolysate was fortified with yeast extract [84].

*Mannheimia succiniciproducens:* *M. succiniciproducens* is a non-spore-forming, facultative, mesophilic, capnophilic, non-motile, and gram-negative bacterium. Genome analysis indicates *M. succiniciproducens* is the closest relative of *A. succinogenes* and share many features with other natural SA producers. The bacterium can efficiently utilize glucose, mannitol, arabitol, fructose, xylose, sucrose, maltose, and lactose, therefore, a promising cell factory for accumulating SA from all major carbon sources abundant in nature [33, 85]. *M. succiniciproducens* is one of the best strains for SA biomanufacturing among SA-producing microorganisms, and SA is produced through the reductive branch of the TCA cycle. Unlike *A. succinogenes*, *M. succiniciproducens* has a complete TCA cycle, indicating that its metabolism may have different and more complex controls for diverting carbon flux towards SA. PEP carboxylation by PEP carboxykinase is a key step for SA accumulation by *M. succiniciproducens* as severe retardation in cell growth and SA production was observed with PEP carboxykinase mutant, which was not the case with PEP carboxylase mutant [86, 87]. The bacterium contains lactate dehydrogenase, pyruvate formate lyase, phosphotransacetylase, and acetate kinase as the major pyruvate dissimilating enzymes generating ethanol, lactic, acetic, and formic acid as byproducts [30].

As mentioned above that SA via reductive TCA cycle requires CO_2_ as co-substrate and supply of reducing equivalents. *M. succiniciproducens* depend heavily on CO_2_ uptake for cellular growth and SA production. There is a very interesting work by Ahn et al. where formic acid/formate (FA) was used as a co-substrate to supply CO_2_ and an additional source of NADH [31]. They found that *M. succiniciproducens* contains a formate transporter, and ^13^C isotope analysis confirmed that bacterium has the ability to metabolize FA via two different routes. The formate dehydrogenase (FDH) mediated route converts FA into CO_2_ which is used for anaplerotic carboxylation reaction catalysed by PEP carboxylase and/or PEP carboxykinase and also generates one NADH [HCOOH + NAD^+^  → CO_2_ + NADH]. The other route makes use of reverse pyruvate formate lyase reaction to transform FA into pyruvic acid [Acetyl-CoA + HCOOH → C_3_H_4_O_3_ + CoA-SH]. The FDH route plays an important role in FA metabolism as the forward reaction of PFL is naturally more favorable. They chose metabolically engineered *M. succiniciproducens* LPK7 strain [85] for the said study which contained inactivation of pathways for byproducts formation including acetate (∆*ack*-*pta*), formate (∆*pfl*), and lactate (∆*ldh*). In the said strain, *pfl* gene was knocked off so that FA could be metabolized only via FDH [31]. Due to the low activity of native FDH in *M. succiniciproducens*, several FDH from different sources were heterologously over-expressed and FDH from *Methylobacterium extorquens* was found to be the best. In all the fermentation experiments, sodium formate was used as a source of FA. The fed-batch culture of recombinant strain with glucose and FA as carbon sources generated 69.8 g/L SA with yield and productivity of 0.93 g/g and 2.79 g/L. h. In addition to glucose, other carbon sources were also investigated for SA accumulation. The co-fermentation with glucose, glycerol, and FA resulted in SA titer, yield, and productivity of 72.0 g/L, 1.03 g/g, and 2.88 g/L. h, respectively, while the combination of sucrose and FA produced 76.1 g/L SA, with the conversion yield and productivity of 4.08 g/L. h. This kind of work is very important in current times, when lots of efforts are being made to decarbonize the atmosphere as FA can be made from direct conversion of C1 gas, such as CO_2_, a potent GHG.

In their next work, Ahn et al. deeply investigated the role of MDH in *M. succiniciproducens* for SA biosynthesis [33].Malate dehydrogenase (MDH), catalysing the reduction of OAA into malate, is the committed step in biosynthesis of SA via the rTCA cycle and plays an important role in directing carbon flux from C3 pathway toward SA biosynthesis. Three types of MDH are cytosolic and mitochondrial ones, localized in different cellular compartments with different characteristics. The cytosolic MDH participates in aspartate–malate shuttle, while mitochondrial ones take part in the oxidative TCA cycle. Therefore, it is anticipated that the cytosolic version is more suitable for SA production via the rTCA cycle. Ahn et al. [33] compared MDH of *M. succiniciproducens* with MDHs from well-known natural and non-natural SA producers, including *A. succinogenes*, *C. glutamicum*, *E. coli*, *S. cerevisiae* (cytosolic, mitochondrial, and glyoxysomal), and *Y. lipolytica* (cytosolic). Among the eight MDHs, they successfully purified four MDH’s (from *C. glutamicum*, *E. coli*, *M. succiniciproducens,* and *Y. lipolytica*), which were compared for reduction of OAA. They found that MDH from *C. glutamicum* exhibited the highest activity, while the lowest activity was surprisingly observed with MDH from *M. succiniciproducens*, an efficient SA producer. Next, they performed kinetic analysis of MDH from *C. glutamicum* (CgMDH) and *M. succiniciproducens* (MsMDH) using OAA and NADH as substrates. The CgMDH exhibited the highest activity at pH 7.0, coinciding well with optimal pH (6.5–7.2) of growth of *M. succiniciproducens* [88, 89], while the maximum activity of MsMDH was observed at pH 9.0, which significantly reduced at acidic pH. The CgMDH showed higher *k*_*cat*_ and *k*_*m*_ than MsMDH in the pH range of 5.0–7.0, resulting in similar catalytic efficiencies (*k*_*cat*_/*k*_*m*_). Both the enzymes exhibited substrate (OAA) inhibition; however, the degree of inhibition was significantly higher in the case of MsMDH than CgMDH, which showed mild inhibition as reflected by inhibition constant *k*_*i*_ values. The high activity and low susceptibility of CgMDH towards substrate inhibition clearly indicate that it will be a better choice for effective SA production in *M. succinicproducens*. Therefore, MsMDH in *M. succiniciproducens* ∆*ldhA* ∆*pta-ackA* was replaced with CgMDH. The fed-batch fermentation of the resulting recombinant strain using glucose and glycerol as carbon sources generated 101.2 g/L SA with yield and productivity of 0.90 g/g and 4.18 g/L. h, respectively. Further improvement in TYP metrics was brought by increasing the inoculum size. The fed-batch culture of recombinant strain coupled with high inoculum dose (OD_600_: 19.3 ~ 8.7 g dry cell weight/L) amassed 134.3 g/L SA with a yield of 0.82 g/g and overall productivity was 10.3 g/L. h. This is one of the best fermentative SA production reported [33].

Recently, cell permeability and transporters have drawn a lot of attention in regard to SA production. In this aspect, the same group conducted membrane engineering study with *M. succiniproducens* PALK in which genes encoding for lactate dehydrogenase (*ldhA)*, phosphotransacetylase (*pta)* and acetate kinase (*ackA*), were already disrupted*.* Two genes, namely, *cti* and *pelB* which encoded for cis–trans isomerase enzyme and signal peptide, respectively, were over-expressed. The enzyme cis–trans isomerase catalyzes the conversion of *cis*-fatty acid to *trans*-fatty acid in the cell membrane, thereby altering its fatty acid composition and impacting the membrane fluidity. When the *cti* gene from *Pseudomonas aeruginosa* was over-expressed, it not only enhanced membrane rigidity of the engineered strain due to high *trans*-unsaturated fatty acids (TUFA) content but conferred low pH and acid tolerance. When the fed-batch experiments were conducted with this engineered strain, a maximum of 84.21 and 97.1 g/L of SA was produced from glucose and glucose + glycerol, respectively, with productivity of > 3.0 g/L/h [44]. Despite the immense potential of all these native producers, rational metabolic engineering, synthetic biology, and evolutionary engineering work for optimizing the carbon flux towards SA, cofactor engineering, eliminating byproducts and negative regulatory circuits, and overcoming end-product toxicity is lacking.

*Corynebacterium glutamicum:* *C. glutamicum* is a fast-growing, non-motile, gram-positive, and facultative anaerobic microorganism. The bacterium is a well-known amino acid-producing industrial organism of the fermentation industry with GRAS status [90]. It can metabolize many carbon sources for its growth and energy supply: glucose, fructose, ribose, sucrose, mannose, and maltose. *C. glutamicum* has been explored for the production of several organic acids, including pyruvic, lactic, α-ketoglutaric, and SA [45]. The bacterium accumulates organic acids, such as lactic acid and SA, under oxygen deprivation, where energy and carbon flux are channelized towards product formation instead of accumulating biomass. In other words, the transition from aerobic to micro-aerobic/anaerobic conditions has a strong impact on organic acid production, where cell growth is arrested under oxygen deprivation, but cells retain the ability to metabolize sugars to organic acids [46, 47]. This shift allows the bacterium to adjust its metabolic behavior through the amplification of genes encoding for glycolytic, fermentative, and reductive TCA cycle [91]. *C. glutamicum* exhibits great potential to overproduce SA [92, 93]. Thus, *C. glutamicum* is an excellent example of growth-decoupled SA production where aerobically grown cells are harvested in the first stage, followed by the transfer of these cells to production vessels for bioconversion of carbon source to SA [48]. Like *A. succinogenes*, *C. glutamicum* synthesizes SA via the reductive branch of the TCA cycle, and anaplerotic enzymes connecting the C3 and C4 pathways are pyruvate carboxylase, PEP carboxylase, PEP carboxykinase, malic enzyme, and oxaloacetate decarboxylase.

*C. glutamicum* is known to produce as high as 146 g/L of SA after genetic modification [46]. Yet state of the art from past one decade reveals that besides understanding the metabolic switches which led to enhanced SA production via gene manipulation, the researchers are now focusing to evaluate the performance of genetically modified *C. glutamicum* for efficient valorization of lignocellulosic sugars to SA.

For instance, Mao et al. [45] attempted to alter the genes of *C. glutamicum* so that it can utilize xylose which is considered a cornerstone for LCB-based biorefinery. *C. glutamicum* cannot metabolize xylose, and to empower xylose utilization, *xylA* (xylose isomerase) and *xylB* (xylulokinase) were outsourced from *Xanthomonas campestris* after screening from *E. coli*, *Paenibacillus polymyxa* SC2, *Streptomyces coelicolor,* and *X. campestris*. Furthermore, plasmids containing pyruvate carboxylase (*pyc*), citrate synthase (*gltA*) and succinate exporter (*sucE*), *xylA* and *xylB* were overexpressed in recombinant *C. glutamicum* strain with deletion of *ldhA*, *ackA*-*pta*, *pqo* and *cat* genes and replacement of the native promoters of *pyc* and *ppc* with the *sod* promoter. The resulting strain exhibited better results on xylose than glucose in terms of SA titer (27.4 versus 24.6 g/L) and yield (0.90 versus 0.81 g/g) with reduced accumulation of pyruvate, which is due to the fact that PTS is not used for xylose transport. For enhancing xylose uptake, transketolase (*tkt*) and transaldolase (*tal*) were overexpressed to divert carbon flux from the non-oxidative pentose phosphate pathway towards the glycolytic pathway. Furthermore, to facilitate sugar transport, the pentose transporter *araE* was outsourced from *Bacillus subtilis* and integrated into the chromosome at *ldh* focus, and with all these changes, SA productivity improved to 2.28 g/L. h. The engineered strain was cultured on different glucose:xylose sugar ratios and found no obvious difference in SA titer (29–32 g/L) and yield (0.97–1.00 g/g) and demonstrated the potential of strain to metabolize a broad range of LCB. The batch cultivation of engineered strain on a sugar mixture containing 81.3 g/L glucose and 40.3 g/L xylose, a ratio consistent with LCB hydrolysate, under anaerobic conditions produced 100.2 g/L SA within 23 h and conversion yield was 0.82 g/g sugar. The carbon loss was attributed to the soaring accumulation of α-ketoglutarate (16.2 g/L), indicating substantial activity of the oxidative TCA arm under anaerobic conditions, which were speculated to maintain the redox balance. Finally, corn stalk hydrolysate containing 71.0 g/L glucose and 30.1 g/L xylose was utilized for SA production, and the fermentation profile was similar to pure sugars in terms of titer (98.6 g/L) and productivity (4.29 g/L. h) and α-ketoglutarate formation (11.6 g/L) as a byproduct. Surprisingly, the yield (0.98 g/g) was 16.3% higher than pure sugars, and authors speculated it could be due to consumption of citric acid/sodium citrate in the culture medium and other sugars in the hydrolysate [45].

A similar approach was adopted by Li et al. in developing *C. glutamicum* by pushing more carbon flux from C3 to C4 pathway towards SA through overexpression of pyruvate and PEP carboxylase, elimination of competing pathways (lactate and acetate) and overcoming the inhibition mediated through end product by overexpression of *Ncgl0275,* as described above [50]. The fed-batch culture of engineered strain on glucose accumulated 117.8 g/L SA with yield and productivity of 0.59 g/g and 1.04 g/L. h, respectively, and acetate as a major byproduct (~ 15 g/L). Further to connect xylose metabolism with central carbon metabolism, two xylose utilization, non-phosphorylative Weimberg [xylose dehydrogenase (*xylBc*), 2-keto-3-deoxy-d-xylonate dehydratase (*xylXc*), 1,4-xylono lactonase (*xylCc*), xylonate dehydratase (*xylDc*), and α-ketoglutarate semi aldehyde dehydrogenase (*xylAc*)] and isomerase [xylose isomerase (*xylA*), xylulokinase (*xylB*), and xylose transporter (*xylE*)] pathways were introduced. Despite the presence of two pathways, only 65% xylose (19.5 g/L from 30.0 g/L) was utilized after 96 h of fermentation, and to troubleshoot it, the culture medium was supplemented with glucose as a rapid energy provider. Although xylose was not fully metabolized, the fermentation of mixed sugars (70 g/L of glucose + 30 g/L xylose) enhanced xylose consumption, and the recombinant strain generated 64.2 g/L SA after 60 h. The fermentation using hydrolysate from CASA (concentrated-alkali under steam-assistant) pretreated corn stover with 67.7 g/L glucose and 21.7 g/L xylose yielded 64.1 g/L SA and 10.1 g/L acetate as byproduct after 72 h [50]. Though TYP metrics were similar to mixtures of pure glucose and xylose, the uptake of xylose was slower due to the presence of inhibitors and limited nutrients.

Most recently, CRISPR–Cpf1 system was used for editing *C. glutamicum* ATCC 13032 so that it can consume fermentable sugars from enzymatic hydrolysate of H_2_O_2_–acetic acid (HPAC) pretreated *Pinus densiflora* [94]. CRISPR–Cpf1 was preferred over CRISPR–Cas9 as the latter could get deactivated due to secretion of various toxic metabolites while the former was more efficient. Gene encoding for lactate dehydrogenase enzyme was knocked out whereas SA transporter was overexpressed. When fed-batch cultivation was performed with the engineered bacterium using 4% hydrolysate, a maximum of 39.47 g/L of SA was obtained in 48 h, with 100% and 73% glucose and xylose consumption, respectively [94].

Two notable genetic engineering approaches are discussed in succeeding paragraphs wherein PTS-defective *Corynebacterium glutamicum* [49] and highly efficient SA tolerant bacterium [48] were designed to accumulate industrially relevant titers of desired end-product. The phosphotransferase (PTS) is responsible for efficient glucose uptake but with a heavy cost with one mole of PEP for every mole of glucose taken up via PTS. This step requires almost half of the available PEP for glucose uptake and phosphorylation; therefore, it significantly limits the amount of SA that can be synthesized as PEP is also a precursor for SA [95, 96]. Zhou et al. knocked off PTS (*ΔptsG*) in *C. glutamicum* to enhance the availability of PEP for SA biosynthesis, which seriously impaired the growth on glucose [49]. To restore the glucose uptake, transcriptional regulator *iolR* was deleted, which exerted positive impacts with higher growth and glucose uptake rates and also caused increment in transcription levels of two myo-inositol transporter (*iolT1* and *iolT2*) and glucokinase (*glk* and *ppgk*) genes. To bring it equal to the wildtype strain, myo-inositol transporter (*iolT1*) and polyphosphate glucokinase (*ppgk*) were overexpressed. The deletion of *iolR* and overexpression of *iolT1* and *ppgk *in a *ΔptsG* background completely restored glucose utilization and improved SA production. The fed-batch culture of recombinant (*ΔptsG ΔiolR ↑ioT1 ↑ppgk*) strain under anaerobic conditions generated 90.8 g/L (769 mM) in 48 h with the consumption of 94.0 g/L (522.2 mM) glucose. The TYP matrices were improved by 11.6%, 32.4%, and 11.2% compared to the control strain, respectively. The results indicate that uncoupling glucose transport from PTS causes improvement in the supply of PEP towards SA through pyruvate and PEP carboxylase anaplerotic reactions, which eventually leads to a substantial increment in SA bioproduction.

Chung et al. investigated end-product toxicity caused by SA with the presence of external 0.25 M SA for *C. glutamicum* strain (*Δldh, ΔpoxB, Δpta–ackA, ΔactA*) with quadruple deletion [48]. They observed IC_50_ values of 0.10 and 0.11 M glucose uptake rate and SA production, indicating impairment of carbon metabolism by extracellular SA. The transcriptomic analysis in the presence of external SA (0, 0.0625, 0.125, and 0.25 M) showed that “*NCgl0275”* was among several down-regulated genes. *NCgl0275* is a homolog of the WhiB family transcriptional protein, which acts as a negative regulator in the oxidative stress response pathway [97]. When *NCgl0275* gene was over-expressed, it not only enhanced glucose uptake but also showed an improvement of 37.7%, 43.2% and 37.9% in SA titer (55.4 g/L), yield (0.53 g/g), and productivity (0.80 g/L. h) in comparison to control strain, respectively, during fed-batch cultivation. Next anaplerotic reactions were targeted for improving pyruvate/PEP to OAA conversion: the native *pyc* (pyruvate carboxylase) gene was substituted with *pyc*^P458S^; the native promoter of *ppc* (PEP carboxylase) was replaced by strong *tuf* promoter and native *pckG* (GTP-dependent PEP carboxykinase) was changed with that of *M. succiniciproducens* under *tuf* promoter. Furthermore, *ptsG* gene was knocked off to divert the PEP/pyruvate pool toward SA biosynthesis. The final engineered strain carrying all these modifications (Δ*ldh*, Δ*poxB*, Δ*pta–ackA*, Δ*actA*Δ*pck*Δ*ptsG* ↑*pycP458* ↑*Ptuf::pckG*↑*Ptuf::ppc* ↑*NCgl0275*) consumed 139 g/L glucose and amassed 152.2 g/L SA under oxygen-limited conditions in fermentation period of 160 h [48].

These studies reaffirm that still there can be number of unconventional genes which can indirectly but significantly impact SA production. Inclusion of transcriptome analysis and integrating its results with gene modification can further open newer avenues for attaining cost-competitive bio-based SA titers.

*Other naturally SA producing microbial strains:* Besides, the conventional naturally SA producing strains discussed in the preceding sections, bioprospecting is still a popular strategy to isolate new SA producing strains. For instance, Szczerba et al. [52] screened rumen samples to isolate bacteria which can produce SA from lactose under anaerobic conditions. From 50 isolates, 26 were able to produce SA from lactose and the best SA producer was identified as *Enterobacter aerogenes* LU-2 based on molecular identification. After optimization of various parameters such as pH, temperature, size of the inoculum, yeast extract etc. when batch studies were conducted with pure lactose and whey permeate, the wild strain produced 51.35 and 57.7 g/L of SA, respectively.

Likewise, Nagime et al. [98], adopted a two-stage strategy to screen SA producing bacterial strains obtained from various sources. In the first stage, the isolated organisms were streaked on agar and incubated under anaerobic conditions. Based on zone of clearance, the organisms were subjected to growth and fermentation and presence of SA was detected by thin layer chromatography (TLC) based on *R*_*f*_ values. Only two organisms isolated from rumen showed positive results in TLC. These bacterial isolates which were phylogenetically identified as *Enterococcus gallinarum* and *Bacillus velezensis,* produced 66.9 and 50.2 g/L of SA in 60 h and 48 h, respectively, when glucose was used as the carbon source. However, when glucose was replaced by low carbon feedstock such as mixture of palm oil mill wastewater and molasses (80:20) and yeast extract was substituted by peptone, the maximum SA titer attained were 73.9 g/L with *E. gallinarum.* Earlier Kunez et al. [99] adjudged the performance of their newly isolated AKR177 strain on pure and crude glycerol (PG and CG). This isolate which belonged to genus *Actinobacteria* under fed-batch cultivation, resulted in maximum accumulation of 117 and 86.9 g/L SA using PG and CG, respectively, when in the second phase MgCO_3_ was replaced by Na_2_CO_3_ as pH regulator and pH was maintained at 7.3.

## Non-native SA producing strains

There are a number of bacterial and yeast host systems which do not produce SA naturally. Some of them generate SA as byproduct but not as main/end product. Since their genome is fully mapped and the expression systems for genetic manipulations are in place, they are often capitalized for producing a variety of industrially important bio-based chemicals including SA. The succeeding section features those engineered microbes (both prokaryotes and eukaryotes), that have been exploited for SA biomanufacturing.

*Escherichia coli:* *E. coli* is a facultative gram-negative bacterium with the ability to assimilate a number of carbon sources, such as glucose, xylose, arabinose, sucrose, glycerol, acetate, etc. Unlike natural SA producers, *E. coli* generates SA as an intermediate but not as an end product. The bacterium can synthesize SA under aerobic as well as anaerobic conditions. Under aerobic conditions, SA is formed through the oxidative branch of the TCA cycle by succinyl-CoA synthetase, which is subsequently transformed to fumarate by succinate dehydrogenase. In other words, SA is formed only as an intermediate under aerobic conditions. That is why wild-type cultures of *E. coli* do not accumulate SA under aerobic conditions. On the contrary, the bacterium undergoes mixed acid fermentation with ethanol, formic, acetic, and lactic as major fermentation products, and in comparison, the amount of SA formed is minor. The major carboxylating enzyme is PEP carboxylase, which catalyses the irreversible conversion of PEP to OAA without yielding ATP. On the other hand, the PEPCK counterpart of PEPC in native producers is reversible, active during gluconeogenesis, and generates ATP. *E. coli* also has a Glyoxylate cycle for SA production, which is active under aerobic conditions [25].

Way back in 2002, Vemuri et al. employed *E. coli* AFP111 strain (∆*pfl* ∆*ldhA*) with one more mutation in *ptsG* gene, part of phosphotransferase system, which renders its reliance on glucokinase for glucose assimilation [53]. Furthermore, pyruvate carboxylase was overexpressed to divert glycolytic flux toward the reductive TCA cycle for SA production. This recombinant strain was employed for SA biosynthesis via dual-phase fermentation to uncouple growth and product formation. The transition time for switching from aerobic to anaerobic conditions was optimized, as SA accumulation is tightly linked to the complex interplay of various pathway enzymes whose expression changes with fluctuation in oxygen levels during the course of fermentation. The optimal transition time led to accumulation of 99.2 g/L SA with a yield of 110% and productivity of 1.3 g/L. h. Since isocitrate lyase is not active under anaerobic conditions, it is believed that the major contribution of SA was from the reductive TCA cycle. The pyruvate metabolism was active in the absence of pyruvate formate lyase even under anaerobic conditions, where pyruvate dehydrogenase is assumed to be absent [51].

Six year later, Jantama et al. used a combinatorial approach of metabolic and evolutionary engineering to design a SA accumulating *E. coli* strain [54]. The biochemical reactions [*ldhA* (lactate dehydrogenase), *adhE* (alcohol dehydrogenase)] serving as primary routes for NADH oxidation and acetate [*ackA* (acetate kinase)] production were eliminated so that cell growth and ATP synthesis remains tightly coupled to SA biosynthesis for NADH oxidation. The strain was metabolically evolved by carrying out pH-controlled fermentation to circumvent the negative impacts of deletions of various genes and allow maximum flux through the SA pathway without perturbations in redox balance, ATP production, and cell growth. Later, *pflB* (pyruvate formate lyase), which is responsible for acetyl-CoA production from pyruvate and *focA* gene which encodes for formate transport, were deleted to eliminate carbon loss and formate as a reductant. This made the strain auxotrophic for acetate under anaerobic conditions and the acetate requirement was compensated through metabolic evolution with possible participation of other routes, such as pyruvate dehydrogenase complex generating acetyl-CoA. During the entire study the strain was metabolically evolved by sub-culturing *E. coli* over 2,000 generations, based on growth-based selection. The anaerobic batch fermentation of evolved recombinant strain (*∆ldhA, ∆adhE, ∆ackA, ∆focA, ∆pflB*) generated 733 mM SA (86.6 g/L) with yield and productivity of 1.41 mol/mol (0.93 g/g) and 0.90 g/L. h, respectively. The cell growth was accomplished in the initial 48 h while SA accumulation continued for 96 h and one-third of SA production was achieved in the absence of cell growth.

However, very recently Liu et al. [55] filed a patent wherein in the first phase three genes, namely, *pflb, focA* and *ldhA,* were knocked out from *E. coli* to reduce formation of two byproducts, namely, formic acid and lactic acid. Later, acetic acid formation was prevented by disrupting the gene (*ptc–ack*) that encodes for phosphotransacetylase–acetate kinase. Two heterologous genes, namely, *pck* and *ptxD* which encoded for phosphoenopyruvate carboxykinase and phosphite dehydrogenase that were obtained from *A. succinogenes* and *Pseudomonas stutzeri*, respectively, were over-expressed. Later, the invention claims the use of recombinant strain for SA production via two-stage fermentation process. In the first stage, aerobic conditions were provided to support bacterial growth and when the bacterial cell density reached a OD_600nm_ of 55–60, the fermentation was switched to anaerobic conditions wherein glucose was fed in a controlled manner under pH–stat conditions. Within 96 h, the strain accumulated 137 g/L SA with yield and productivity being 1 g/g glucose and 1.43 g/L/h, respectively. Furthermore, the inventors have claimed no formate or lactate formation and even acetic acid production reported was ≤ 2 g/L [55].

Glycerol is also an interesting carbon source for microbial cell factories, and being a reductive substrate, it turns out to be a better substrate than glucose for SA, where SA production is limited by the availability of reducing equivalents. That is why SA yield on glycerol (1.0 mol/mol glycerol) is higher than glucose (0.86 mol/0.5 mol glucose). In 2018, *E. coli* strain MLB (-*ldh*,-*pflb*) was genetically manipulated by over-expressing *pck* gene which encodes for phosphoenolpyruvate carboxykinase with an intent to fix CO_2_ during glycerol assimilation [56]. Later, the recombinant strain was evaluated in a two-stage fermentation process where aerobic and anaerobic conditions were maintained, respectively, in first and second phase. When three different types of glycerol were used as the carbon source, the maximum SA titers attained were 72.67 g/L, with pure glycerol. With crude glycerol, the titers achieved were merely 5.9 g/L. However, when the crude glycerol was subjected to activated charcoal and used as feedstock, it led to accumulation of 66.78 g/L SA, highlighting the important role of pretreatment for better glycerol assimilation by engineered *E. coli* and successive valorization to SA.

In the next year only, Yu et al. performed detailed metabolic engineering work on *E. coli* for glycerol-based production of SA [57]. The anaerobic fermentation of glycerol suffers from redox imbalance and limited energy supply to support cell growth, and SA export and energy requirement goes up as more and more SA accumulates. In *E. coli*, glycerol is oxidatively metabolized by two different routes; in one pathway, glycerol is oxidized to dihydroxyacetone (DHA) by glycerol dehydrogenase (GldA) [glycerol + NAD^+^  → DHA + NADH] followed by phosphorylation of DHA to dihydroxyacetone phosphate (DHAP) mediated by PEP-dependent DHA kinase (DhaKLM) [DHA + PEP → DHAP + pyruvate]. The problem with this route is that it takes away PEP, a precursor for the biosynthesis of SA, and negatively impacts SA formation. In the second route, glycerol is phosphorylated to glycerol-3-phosphate (glycerol kinase; GlpK) [glycerol + ATP → glycerol-3-phosphate + ADP] which is oxidized to DHAP by menaquinone dependent glycerol-3-phosphate dehydrogenase (GlpABC) [glycerol-3-phosphate + menaquinone → DHAP + menaquinol]. This pathway has a shortage of energy. The metabolic engineering work was aimed at generating two NADH from the conversion of glycerol to PEP instead of one NADH and menaquinol, enhancing the availability of PEP for SA production and improving the energy supply. The engineered strain contained deletion of *ldhA* (lactate dehydrogenase) and *pflB* (pyruvate formate lyase) and *ptSI* gene and overexpression of galactose permease (GalP) as an alternative to glucose utilization pathway and PEP carboxykinase [PEP + CO_2_ + ADP → OAA + ATP] to replace native PEP carboxylase, an ATP generating biochemical reaction. Furthermore, the deletion of *glpK* and *dhaKLM* genes disabled the growth on glycerol, and the introduction of exogenous ATP-dependent dihydroxyacetone kinase (DhaK) allowed the metabolism of glycerol through the GldA–DhaK pathway. The substrate-level phosphorylation is the main or only source of energy under anaerobic metabolism. In the case of SA, during the conversion of fumarate to SA, NADH dehydrogenase (NDH-1) transfers electrons from NADH to menaquinone to form menaquinol, which is used to reduce fumarate to SA. This reaction also pumps out 4 protons [100], and the proton motive force generated provides energy in the form of ATP, which is another source of energy in addition to substrate-level phosphorylation [101]. Thus, the GldA–DhaK pathway [glycerol + CO_2_ → SA + ATP + 4H^+^_out_] can provide more energy and better SA production in comparison to existing pathways in *E. coli*. All these changes helped secure sufficient ATP, PEP, and NADH supplies for enhanced SA production while maintaining redox balance. The designed recombinant *E. coli* strain with all these modifications accumulated 483 mM (57 g/L) SA in 96 h with a conversion yield of 0.92 mol/mol.

As discussed earlier, there is an increasing trend of exploring the application of engineered microbes to assimilate low-cost feedstocks such as lignocellulosic biomass and valorize them to industrially important bio-based chemicals including SA. For instance Liang et al. metabolically engineered *E. coli* with deletion of *pflB*, *ldhA*, *ppc,* and *ptsG* and overexpression of ATP forming PEPCK [58]. The insufficient ATP supply during SA production is a bottleneck for achieving high yield and productivity. This situation is exacerbated in the case of xylose, where ATP supply is lower than glucose as substrate [102] and the presence of PEPCK can contribute towards alleviating it. The SA accumulated under anaerobic conditions during repetitive fermentation by recombinant strain on glucose and xylose as carbon source were 32–35 and 24–25 g/L with a conversion yield of 0.94–0.97 and 0.98–1.03 g/g, respectively. The changes in SA production metrics from the first to third stages were marginal. Similar results were achieved during co-fermentation on a mixture of glucose and xylose, and simultaneous consumption of two sugars was achieved due to mutation in *ptsG*. It was speculated that improved xylose assimilation was achieved due to high ATP supply from glucose fermentation, complementing less ATP production from xylose metabolism. They also made use of sugarcane bagasse (60% glucose and 30% xylose) and corn stover (10% glucose and 80% xylose) hydrolysate for repetitive SA fermentation. In the case of sugarcane bagasse hydrolysate, the SA titer achieved was 83 g/L in 36 h of three repetitive stages with a conversion yield of 0.87 g/g, while it was 61 g/L with a yield of 0.92 g/g for corn stover hydrolysate. The rate of fermentation was slower in corn stover than the sugarcane bagasse hydrolysate, and this could be a difference in ATP levels as activities of key enzymes and NADH/NAD^+^ exhibited no differences.

Recently Zhu et al. [59, 60] assessed the performance of their *E. coli* (FZ661T) on galactose rich feedstocks and wood hydrolysate, respectively, taking pure (mixed) sugars as control. This genetically modified (GM) strain was obtained by disrupting several competing pathways which led to byproducts formation, activating glyoxylate pathway and facilitating galactose utilization by altering several genes of *gal* operon. A two-stage fermentation process was set up where cell growth was achieved in aerobic phase and the pH was not controlled. However, when the conditions were switched to anaerobic conditions, the pH was controlled above 6.8 and it represented SA production phase. In a fed-batch process, the GM strain produced 95.8 and 74 g/L SA when the feed was mixed sugar and galactose fortified soybean molasses hydrolysate, respectively [59].When the same strain was tested for wood hydrolysate in a batch fermentation it produced 54.5 g/L SA while the pure sugars (xylose + glucose) in a fed-batch fermentation produced as high as 107 g/L SA [60].

In yet another study an engineered *E. coli* strain KJ122 was for the first time tested for SA production through simultaneous saccharification and fermentation (SSF) using alkali pretreated rice straw as its feedstock [61]. This strain was obtained by disrupting several genes in the engineered *E. coli* strain obtained by Jantama et al. [54], which led to high acetate, pyruvate and malate formation. When the robustness of the said strain was tested under SSF conditions, unlike batch which produced 69.8 g/L SA, fed-batch conditions resulted in accumulation of 103.1 g/L of SA [61]. Earlier using the same organism, the group reported the production of 98.6 g/L of SA under fed-batch SSF when the feedstock was cassava pulp [62].

*Other bacterial strains:* In the latest reports, an engineered strain of *Klebsiella oxytoca* was developed in which genes, namely, *adhE*, *pta–ack*, *ldhA*, *budAB* and *pflB,* were disrupted [63]. It was obtained from M5A1 strain which grows on diverse substrates, has no specific growth requirements, qualifies biosafety aspects and whose metabolic engineering tools are available. However, the engineered strain did not produce any SA after manipulation so it was metabolically evolved over 6000 generations and later tested for SA production under anaerobic conditions. The evolved strain produced a maximum of 82.88 and 57.5 g/L SA when the feedstock was glucose and sugar molasses, respectively. The transcriptome analysis revealed that in the adapted strain, the expression of two genes particularly *pck* and *tdcE* was elevated whereas several genes such as *pykA*, *acs*, *poxB*, *tdcD* and *pdhR* were downregulated. This study thus opens new avenues for lesser known bacterial strains for SA production [63].

Recently Thoma et al. [64] selected *Vibrio natriegens* for SA production owing to its fully annotated genome, expression systems in place, rapid biomass production under resting stage and more prominently harboring genes for SA production both under aerobic and anaerobic conditions. Competing pathways for lactate, acetate, ethanol and formate production were inactivated by disrupting the genes encoding for their formation. Later, *pyc* gene from *C. glutamicum* was overexpressed by chromosomal integration to enhance anaplerotic flux. Under anaerobic conditions, the modified strain produced SA with a molar yield of 1.46 and exhibited high biomass formation. In a zero-growth bioprocess which involved use of resting cells, the 60.4 g/L of SA was produced in merely 7 h. This is one of the best reports where the SA productivity was as high as 8.62 g/L/h [64].

Despite several investigations, bacterial fermentations are quite sensitive to pH fluctuations and unable to grow effectively at low pH values (< 5.0). If the end product is an organic acid, then a pH control (near to 6.0–7.0) throughout the fermentation is necessary. The bacterial fermentation with titration agents results in SA in the form of salt rather than the acid form, which complicates the DSP as salt need to be acidified to bring it back to acid form, making it expensive. DSP is an expensive unit operation and contributes to 60–70% of the total cost in the case of SA. Therefore, fermentation at low pH without a neutralizing agent, where SA can exist in acid form, is highly desirable [57]. In comparison, yeasts are the potential host to produce organic acids as they are naturally adapted to grow under low pH. As a result, low pH tolerant yeast-mediated fermentation often simplifies the DSP and reduce the overall production cost of bioprocess. Following yeast systems have been widely explored for SA production:

*Yarrowia lipolytica:* *Y. lipolytica* is a non-conventional, safe, and robust yeast. *Y. lipolytica*, a fascinating microorganism with amazing metabolic flexibility, can robustly metabolize a large variety of substrates, including hydrophilic (glucose, fructose, glycerol, ethanol, acetate) as well as hydrophobic carbon sources (alkanes, fatty acids, and oils) [103]. The yeast is regarded as non-pathogenic and is categorized as GRAS by the FDA (Food and Drug Administration, USA). *Y. lipolytica* does not produce SA naturally, and in initial reports, SA has been synthesized via a semi-synthetic route where α-keto glutaric acid accumulated by yeast was converted into SA with a chemical decarboxylation by H_2_O_2_: α-ketoglutaric acid + H_2_O_2_ → SA + CO_**2**_ + H_2_O [103–106]**.** *Y. lipolytica* VKM Y-2412 strain was cultured with ethanol as a carbon source and supplemented with 100 mM H_2_O_2_ which did not affect cell viability. H_2_O_2_ was added gradually, and during the entire cultivation, 580 mM H_2_O_2_ was added. The SA titer achieved after 8 days was 63.4 g/L with a yield of 58% [104]. Similar results were obtained (69 g/L SA) using the same approach when ethanol was replaced with rapeseed oil [105].

Succinate dehydrogenase (SDH) is the key enzyme for SA production through the oxidative TCA cycle, and it is expected that a SDH deficiency would allow SA accumulation. Yuzbashev et al. created mutant strains of *Y. lipolytica *by inactivating the subunits of SDH, *SDH1,* and *SDH2* [107]. The growth of the mutant strains was impaired on glucose but grew and accumulated SA on glycerol. Being more reduced than traditional carbohydrates, glycerol generates more reducing equivalents and consequently extra ATP molecules, which would be really crucial in the case of a truncated TCA cycle. The strain with a mutation in *SDH2* manufactured 45.5 g/L SA with CaCO_3_ as a buffering agent. Using the same approach, Gao et al. inactivated *SDH5* in *Y. lipolytica* and optimized fermentation media and culture conditions [65]. The recombinant strain accumulated 160.2 g/L SA from crude glycerol during fed-batch cultivation with a conversion yield of 0.40 g/g with acetate as the main byproduct. The presence of acetate not only affected cell growth but also diminished the SA yield. In their next study, they identified that the enzyme acetyl-CoA hydrolase (*ach*) hydrolysing acetyl-CoA to acetate is responsible for the overflow of acetate [66]. The enzymatic analysis revealed that it has much higher acetate: succinate CoA-transferase activity (1.89 U/mg) than the hydrolase one (0.03 U/mg). Therefore, besides generating acetate, the enzyme would also reduce SA yield through the formation of succinyl-CoA with its transferase activity (SA + Acetyl-CoA → Succinyl-CoA + Acetate). To curb acetate production, the *ach* gene was knocked out in the SDH-negative *Y. lipolytica* strain, which not only restored cell growth and almost eliminated acetate accumulation (7.5 to 0.2 g/L) but also caused significant enhancement in SA production. However, with the elimination of acetate, a dramatic increase in pyruvate accumulation was observed. The elimination of acetate resulted in the piling up of acetyl-CoA, causing feedback inhibition of pyruvate dehydrogenase, leading to pyruvate accumulation. To divert pyruvate towards SA key enzymes of oxidative TCA, reductive carboxylation and glyoxylate cycle [PEPCK, PYC, citrate synthase, aconitase, succinyl-CoA synthetase beta subunit (SCS2), isocitrate lyase and malate synthase] were overexpressed. The overexpression of PEPCK and/or PYC from *Saccharomyces cerevisiae* and *Y. lipolytica*, respectively, made a notable improvement in SA titer along with considerable malate generation, and pyruvate accumulation was completely stopped. The best results were obtained with recombinant *Y. lipolytica* PGC202 strain overexpressing PEPCK and SCS2 with deletion of ACH and SDH5. The strain amassed 110.7 g/L SA in 138 h with a yield of 0.53 g/g. The pH was not controlled, and the final pH at the end of fermentation was 3.4. This report confirmed the superiority of PEPCK over PYC while diverting the carbon flux from the C3 pathway toward SA production. One of the reasons for this could be due to the supply of additional ATP by PEPCK which may be beneficial to the SDH-negative mutant strain.

Glucose is the most preferred carbon source for industrial microbial fermentations and is also abundant in renewable feedstocks, such as lignocellulosic biomass, food waste, etc. It has been found that the inactivation of SDH has led to insufficient glucose metabolism in *Y. lipolytica* while glycerol assimilation was intact. The truncation of SDH causes inhibition of the conversion of SA to fumaric acid leading to reduced regeneration of reducing equivalents (FADH_2_), and as a result, less ATP is synthesized via oxidative phosphorylation. Furthermore, the export of SA is an energy-expensive process that aggravates ATP deficiency, and this inadequate ATP has been speculated as the reason for the loss of ability of SDH-deleted mutants to grow on glucose [108]. There are some reports in the last 5 years dealing with this problem of the inability of *Y. lipolytica* to grow on glucose. Yang et al. performed the adaptive evolution of *Y. lipolytica* via cell immobilization using cotton absorbent to restore the glucose metabolism, which resulted in significant improvement in glucose uptake rate [67]. The batch fermentation of the evolved strain yielded 65.7 g/L SA with yield and productivity of 0.50 g/g and 0.69 g/L. h, respectively. In a recent study by Jiang et al., optimal combinations of three SA biosynthetic pathways, glyoxylate, oxidative, and reductive TCA cycle, coupled with efficient transport of synthesized SA resulted in high-level SA production by *Y. lipolytica* [68]. The SA biosynthesis in *Y. lipolytica* takes place in mitochondria, and to achieve extracellular secretion, SA must be transported across the inner mitochondrial membrane and cell membrane. The final SA titer obtained is highly dependent on the efficiency of the transport process. The increase in efflux of the end product not only alleviates feedback inhibition and cellular toxicity byproduct but also pushes the equilibrium in the forward direction. To this end, several mitochondrial carriers (MCs) and C4-dicarboxylic transporters were screened to smoothen the transport of SA across the inner and outer membrane. Five MCs were selected, and the best SA production (23.6 g/L and 0.62 g/g glucose) was observed with strain overexpressing mitochondrial dicarboxylate transporter YlDic. Among membrane transporters screened, overexpression of five caused improvement in SA production, and the highest titer and yield were achieved with endogenous YlMae1 and SpMae1 from *Schizosaccharomyces pombe*. Since SpMae1 does not use proton motive force and being energetically less expensive, it was preferred over YlMae1. For diverting glucose carbon towards SA and enhancing its biosynthesis, fumarate reductase encoding gene *TbFrd* from *Trypanosoma brucei* (reductive TCA cycle), endogenous succinyl-CoA synthetase β subunit encoding gene*YlScs2* (oxidative TCA cycle), isocitrate lyase *YlIcl*, malate synthase *YlMls*, and mitochondrial citrate transporter *YlYhm2* (glyoxylate cycle) were overexpressed in *Y. lipolytica* and the strain was designated as PGC62–SYF. The introduction of C4-dicarboxylic acid transporter SpMae1 from *S. pombe* in PGC62–SYF caused further increment in cell growth and SA accumulation, and no improvement was noticed with expression of YlMae1, while the combined overexpression of YlMae1 and SpMae1 in PGC62–SYF resulted in reduced SA production. The fed-batch culture of the strain carrying a simultaneous expression of three SA biosynthetic pathways and cell membrane transporter SpMae1 (PGC62–SYF–Mae) accumulated 101.4 g/L SA from glucose with a yield of 0.37 g/g and productivity of 0.70 g/L. h. This is the highest SA titer achieved with a yeast host using glucose till date [68].

*Saccharomyces cerevisiae:* *S. cerevisiae*, the modern workhorse of industrial biotechnology, is the most well-characterized and thoroughly researched eukaryote. The high acid resistance and osmotolerance of yeast are major advantages over bacterial hosts for SA production, making neutralization cost dispensable and enormously facilitating DSP. SA is not a major product from the metabolism of *S. cerevisiae,* but rewiring of yeast can lead to redirection of C2 (ethanol and acetate) and C3 (glycerol and pyruvate) overflow metabolites towards SA. But at the time, we should be mindful that carbon in yeast prefers to flow to ethanol rather than SA [72]. In *S. cerevisiae*, both oxidative and reductive TCA cycles have been exploited for SA production. The reductive TCA cycle, operated under microaerobic and anaerobic conditions, is thermodynamically not feasible and associated with low activity in *S. cerevisiae*. Raab et al. attempted the oxidative route for SA production by *S. cerevisiae,* and to this end, two enzymes, succinate dehydrogenase (*SDH1* and *SDH2*) and isocitrate dehydrogenase (*IDH1* and *IPH2*), were inactivated to direct flux towards glyoxylate cycle to obtain SA as end-product [29]. These disruptions did not lead to serious growth constraints, and the engineered strain accumulated 3.62 g/L SA on glucose in the shake flask with a conversion yield of 0.11 g/g and productivity of 0.022 g/L. h. In another report by Yan et al., *S. cerevisiae* was engineered to obtain SA via the reductive TCA cycle [69]. Four enzymes [*PYC2* (pyruvate carboxylase), *MDH3R* (malate dehydrogenase), *FumC* (fumarate hydratase), *FRDS1* (fumarate reductase)] were overexpressed in a *pdc* (pyruvate decarboxylase) and *fum1* (fumarase) deficient strain of *S. cerevisiae*. Pyruvate decarboxylase is a major pyruvate consuming enzyme in *S. cerevisiae,* and *pdc*-deficient strain lacks the ability to perform alcoholic fermentation, while FUM1 catalyse the irreversible conversion of fumarate to malate, a major obstacle. Another challenge with the reductive SA pathway is the continuous availability of reducing power due to imbalance in the upper and lower pathways and requires inputs in the form of additional electrons in the form of NADH. This becomes more challenging in *S. cerevisiae,* where NADH is largely taken away for glycerol formation, and the situation is exacerbated in a *pdc*-deficient strain in the absence of ethanol production, resulting in even higher concentrations of glycerol. To overcome this, GPD1 (*gpd1; glycerol 3-phosphate dehydrogenase*) was deleted to block the glycerol pathway. After all these changes, *S. cerevisiae* accumulated 8.1 g/L SA with a yield of 0.26 g/g. Furthermore, they optimized nitrogen, biotin, CO_2_, and pH levels. The batch bioreactor cultivation of engineered strain at a pH of 3.8 and CO_2_ level of 10% with optimized concentrations of biotin and urea resulted in 13.0 g/L SA in 120 h, and the conversion yield was 0.14 g/g [69].

Glycerol being more reduced than traditional carbohydrates such as glucose and higher reducing power of glycerol can be exploited for the production of reducing metabolites requiring more electrons. Therefore, glycerol-based SA production via reductive TCA cycle is carbon dioxide fixing and redox neutral pathway [C_3_H_8_O_3_ (Glycerol) + CO_2_ → C_4_H_6_O_4_ (SA) + H_2_O]. For maximum exploitation of reducing power to generate reducing metabolites in maximum yields, the electrons coming from the oxidation of glycerol must be conserved in the form of cytosolic NAD(P)H to make them available for reduction of OAA to SA rather than transfer to the respiratory chain. To achieve this, the FAD-dependent pathway in *S. cerevisiae* for glycerol catabolism was replaced with the DHA pathway comprising glycerol dehydrogenase and dihydroxyacetone kinase. The synthetic NAD^+^-dependent route cassette was integrated into the genome at *GUT1* locus via CRISPR–Cas9, thereby abolishing the native FAD-dependent pathway. The engineered strain exhibited a maximum specific growth rate of 0.26 h^−1^ on glycerol [70]. In their next study, furthermore, three enzymes [endogenous malate dehydrogenase (*MDH3*), heterologous fumarase (*fumR*), and fumarate reductase (*FRDg*)] converting OAA into SA were integrated into genome along with additional expression of the heterologous dicarboxylic acid transporter DCT-02 from *Aspergillus niger* [72]. For locating MDH and FRDg into the cytosol, the peroxisomal targeting signals were removed from the proteins. The batch culture of the engineered strain accumulated 10.7 g/L SA from glycerol in 168 h, and the conversion yield was 0.22 g/g. SA is an intermediate of glyoxylate cycle, and key enzymes of the pathway are highly upregulated when *S. cerevisiae* is cultured on glycerol as the sole carbon source [109]. The deletion of isocitrate lyase (*ICL1*) reduced the SA titer from 10.7 to 2.9 g/L suggesting a major contribution of the glyoxylate cycle towards SA production. Thus, the highest SA concentration was enabled by the combined activity of both reductive TCA and endogenous glyoxylate cycle. Malubhoy et al. have created second generation SA-producing *S. cerevisiae* strain by changing the design of the expression cassettes for the reductive TCA cycle and also investigated the impact of overexpressing pyruvate carboxylase and addition of CaCO_3_ [71]. A notable improvement was noticed when the culture medium was supplemented with CaCO_3_, providing bicarbonate ion, which acts as co-substrate for pyruvate carboxylase mediated biochemical reaction and might be rate limiting step in designed strain with the optimized reverse TCA pathway. The strain assimilated all the available glycerol without ethanol formation and continued accumulating SA until a titer of ~ 35 g/L was reached after 96 h of cultivation, however, the highest conversion yield of 0.60 g/g was achieved at 72 h. After 72 h, 53.2% of electrons available in glycerol metabolized were conserved in dicarboxylic acid (SA + MA). Malic acid (MA) was obtained as a byproduct, and after 96 h, SA titer reduced while MA concentration was enhanced concomitantly. The other two notable changes in the new strain were that, unlike the first generation base strain, the glyoxylate cycle does not contribute towards SA formation, and the optimized reverse TCA pathway strongly pulls carbon towards fermentative SA production through the redox-balanced pathway. Second, the complete elimination of CO_2_ loss via net CO_2_ consumption was confirmed by off-gas analysis during the active production phase. All these results discussed above indicate the potential of *S. cerevisiae* to act as a cell factory for SA production, however, results are far behind than *Y. lipolytica,* and more work needs to be done for industrial-scale production of SA like ethanol.

*Issatchenkia orientalis* Recently, Tran et al. [73] genetically modified an unconventional low pH tolerant yeast, namely, *Issatchenkia orientalis* in which genes for rTCA cycle were already overexpressed. Further end-to-end process for SA production was demonstrated at pilot scale followed by techno-economic assessment (TEA) and life cycle analysis (LCA) of the entire process. When the codon optimized gene encoding for dicarboxylic acid transporter from *S. pombe* (*SpMAE1*) was overexpressed followed by knocking out of *gpd* (glycerol-3-PO_4_-dehydrogenase), *pdc* (pyruvate decarboxylase) and *g3473* (dicarboxylic acid importer), the strain produced 42 g/L SA in SC-URA medium with 50 g/L of glucose and 20 g/L of glycerol. Further, strain engineering involved overexpression of *Pichia angusta* derived *GDH* (glycerol dehydrogenase) and endogenous *DAK* (dihydroxyacetone kinase), which enhanced glycerol consumption. Thereafter, deletion of *g3837* gene encoding for hexokinase, relieved the engineered strain from catabolite repression exerted by glucose. When the said strain was tested at bench-scale under fed-batch conditions, it produced 109.5 and 104.6 g/L of SA from glycerol + glucose and sugarcane juice, respectively. At pilot-scale (30 L working volume) and under batch conditions, 63.1 g/L SA was produced. Later, the group purified the SA via two-stage vacuum distillation and crystallization method with overall yield being 64% from low-pH fermentation (pH-3.0). Considering pilot-scale scenario as base-case, the minimum selling price for SA at neutral pH and low pH fermentation was found to be US $ 1.17 and US $ 1.05/kg, respectively. The LCA study revealed that for the said process, GWP_100_ which represents 100-year global warming potential was found to be 1.67 kg CO2-eq./kg while fossil energy consumption (FEC) was − 0.21 MJ/kg. This is the first study where such high SA titers were obtained during low pH (3.0) fermentation.

## Downstream processing (DSP) of bio-based SA

**Fig. 3:**
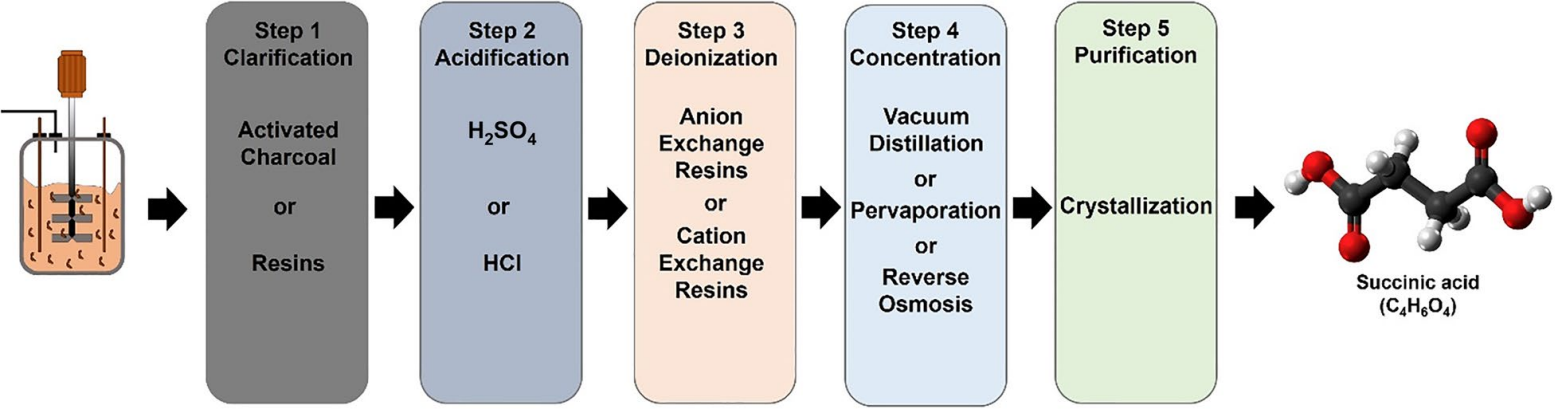
Sequential extraction and crystallization of SA from fermented broth

**Fig. 4:**
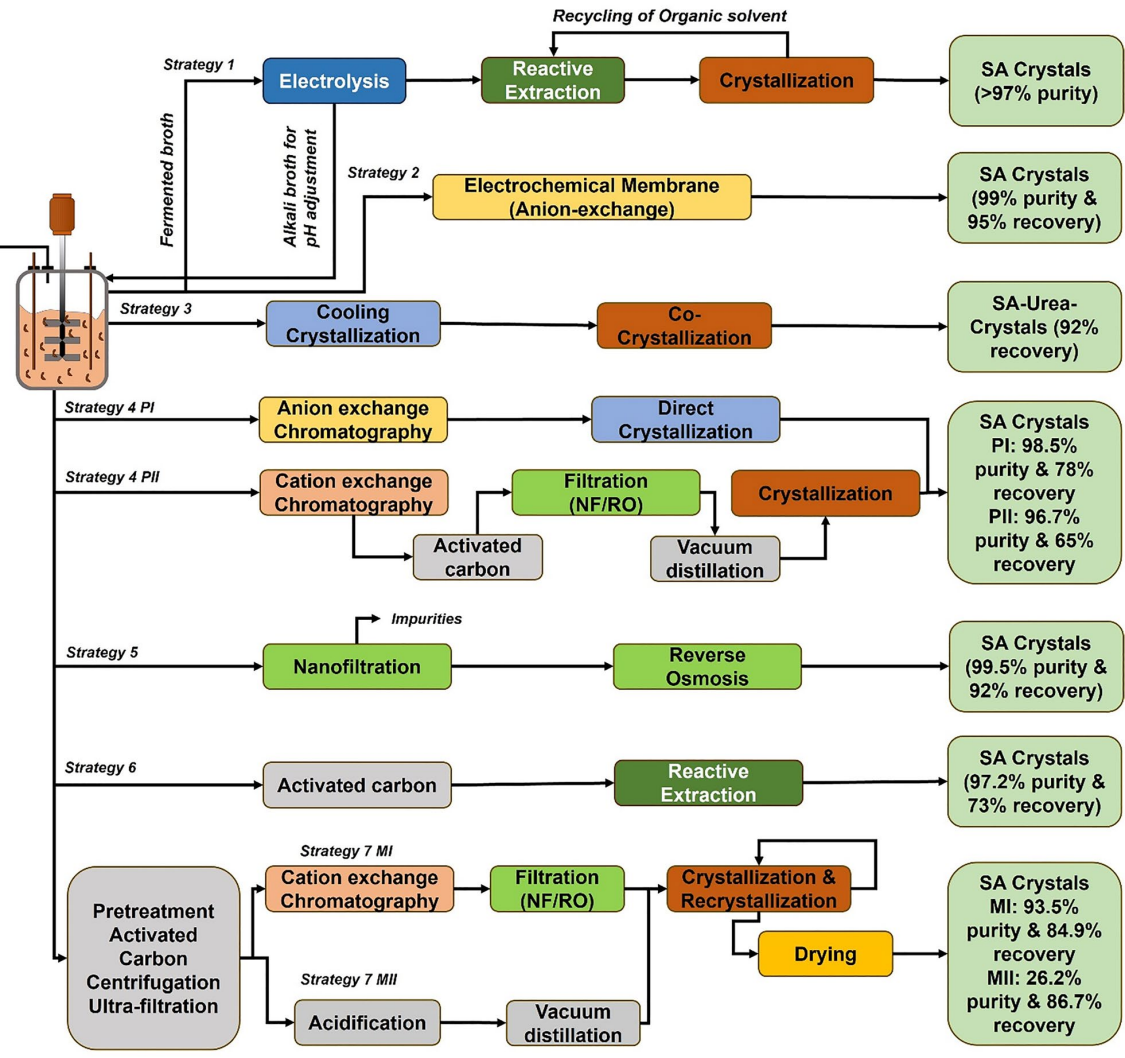
Strategies for downstream processing of SA [19, 67–70, 72, 108]. *PI*: Process 1, *PII:* Process 2, *MI*: Method 1, and *MII*: Method 2

Generally, the DSP of fermentation-derived compounds encompasses several unit operations for the separation, recovery, and purification of the targeted end product. Figure 3 depicts the schematics for the DSP of SA. Post-fermentation, the process involves the separation of microbial cell biomass via centrifugation or filtration, followed by activated charcoal treatment, which leads to clarification and de-coloration of SA-rich supernatant. The supernatant is then subjected to acidification prior to purification techniques (ion exchange, adsorption chromatography, reactive extraction, membrane filtration, distillation, etc.) to obtain the SA in pure form. Once the product is purified and crystallized, SA is subjected to drying, often via techniques like spray drying or freeze-drying, to remove the remaining moisture. Quality control assays are conducted at multiple stages to monitor product purity and consistency. Finally, the purified SA is packaged for storage or distribution, ensuring its quality and stability. Depending on the product purity, SA is used further in various applications, including pharmaceuticals, chemicals, and food products. The literature survey shows various separation techniques like reactive extraction and membrane separations employed for the recovery and purification of SA from simulated solutions [110]. However, the real fermentation broth is usually a complex mixture of other acids, impurities, residual sugars, proteins, polysaccharides, and various soluble and insoluble components [111]. The optimal DSP ensures that the fermentation-derived compounds are obtained at high yield (Y) and high purity (P) suitable for commercial use (Table 3). The conventional processes used for separation and purification of SA from petrochemical or biological routes are calcium precipitation (Y:13%; P:81%), direct crystallization (Y:57%; P:90%), salting out (Y:50%; P:86%), or reactive extraction (Y:73%; P:97.2%) [112]. Even the first commercial biobased SA production venture, BioAmber, utilized the traditional route. The low SA yield in DSP is a key challenge for developing commercial technologies that are non-tedious, energy-efficient, and cost-effective. The following section describes the most recent processes employed for the recovery of high-purity SA from the simulated and fermented broth (Fig. 4).

**Table 3:** List of DSP techniques, their advantages and limitations for separation and recovery of SA

Technique	Advantages	Limitations
Filtration	Effectively and efficiently remove the solid impurities	Membrane fouling
Chromatography	Selective separation of SA with lower impurities	Low yields and selectivity
Adsorption	High selectivity to SA in complex mixtures	Low yields and selectivity
Precipitation	Cost-effective, scalable, and ease of use in SA separation from fermented broth	High energy consumption and substantial salt byproducts
Electrodialysis	Selective separation of succinate ions from broth without acidification	Energy consumption and membrane fouling
Reactive extraction	Efficient separation and purity of SA from broth	Selection of suitable extractant, diluent, and low extraction due to fermentation impurities
Aqueous two-phase extraction	Selective extraction and energy efficient concentration of SA from fermented broth	Selection of suitable extractant, diluent, and low extraction due to fermentation impurities
Crystallization	High purity of SA from the soluble impurities in the aqueous phase	Low yields and require additional purification processes

*Strategy 1:* An integrated multi-phase electrochemical pH shift-based extraction and crystallization strategy was implemented for the separation and purification of SA to achieve high yields and recovery from aqueous solutions. Initially, the pH of the solution containing SA, was adjusted to a neutral pH above the pKa value, then introduced into the first anode chamber, where water electrolysis lowers the pH and increases the fraction of protonated SA. Subsequently, the SA is extracted from the acidified broth into an organic phase. The remaining broth is directed to a cathode chamber coupled to the anode chamber, where OH − ions from water electrolysis raise the pH, allowing for recycling back to the fermenter for pH control without the need  of chemicals for pH adjustments. The loaded organic phase undergoes back-extraction in another cathode chamber, aided by OH − ions, resulting in increased concentration of SA in the aqueous phase for crystallization. The concentrated SA solution is then sent to an anode chamber, where H^+^ ions induce crystallization, yielding solid SA crystals with > 97% purity. Integrating these electrochemical steps minimizes the generation of salt waste, chemical requirement, and allows for continuous processing, offering the promise of both environmentally friendly production and economic benefits through flexible energy management. Further optimization and testing with real fermentation broth are suggested for future work [113].

*Strategy 2:* In the year 2023, a novel electrochemical membrane bioreactor (EMB) was designed where an integrated approach for SA production and its *in-situ* separation has been described using an organic fraction of municipal solid waste (OFMSW) as a feedstock [19]. In the said EMB, an anion exchange membrane (AEM) in the anode compartment facilitated SA separation without the need for centrifugation and acidification stages. The process involved fed-batch fermentation of OFMSW using genetically modified *Y. lipolytica* PSA02004 in a 6.7 L EMB. Two strategies were tested for maximizing SA production and its recovery. In the first strategy, the pH of the medium was maintained at 6.0 and the electrolysis was initiated after 30 h, the maximum SA production reached 47.5 g/L and its extraction flux was 76.6 g/ m^2^h^−1^. In the second strategy,  a two-stage pH regulation strategy was adopted, wherein the pH of the medium was reduced from 6.0 to 5.5 in 30 h and simultaneously electrolysis was initiated, then SA titers reached 66.7 g/L and its extraction flux improved by 17.5%, peaking at 90 g/ m^2^h^−1^. The coulombic efficiency of SA also improved from 56.8% to 66.2%. Moreover, the recirculation of the fermentation broth in the cathode compartment reduced NaOH consumption (35.4%) for pH control. When the solution in the anode compartment was subjected to activated carbon, filtration, vacuum evaporation, crystallization, and drying, SA crystals of 99.95% purity with 95% yield were obtained [19].

*Strategy 3:* An innovative and environmentally friendly approach was adopted for the separation and purification of SA from the fermentation broth of *E. coli*. The process involves two main steps: cooling crystallization and co-crystallization with urea. In the first step, cooling crystallization is employed to separate SA from the fermentation broth, resulting in a recovery rate of 73.4% and a purity of over 99% under optimized conditions of 8 ℃, 4 h, and pH 2.0. This step efficiently removes impurities while maintaining a high SA concentration. In the second step, urea is added to the remaining solution, and co-crystallization is carried out at 4 ℃ for 12 h, achieving a high recovery rate of 92.0%. The resulting SA–urea co-crystal can be further processed to synthesize succinimide with a yield exceeding 80%. This integrated strategy ensures efficient recovery of SA and produces valuable intermediate products, demonstrating its potential for sustainable and cost-effective SA production while reducing environmental impact [114].

*Strategy 4:* The study by Omwene et al. aimed to recover SA from fermentation broth using two different downstream purification processes [115]. The process I involved chromatographic separation with Amberlite IRA900 Cl anionic exchange resin, followed by direct crystallization. Process II included a sequential combination of cationic exchanger, activated carbon, nanofiltration (NF)/reverse osmosis (RO) membrane, vacuum distillation, and crystallization. In Process I, SA was selectively eluted last from the anionic resin column after the removal of lactic acid, acetic acid, and formic acid. The highest chromatographic separation efficiency for SA was 69.3%. In Process II, various purification steps were employed to remove impurities and concentrate SA. The NF90 membrane was used, which showed different rejections for SA, lactic acid, formic acid, and acetic acid at different pH levels, with the highest rejections achieved at pH 6.8. Subsequent double passes through RO with BW30 or HP membranes achieved a high retention rate of 95.9% for SA. The study reported SA purity of 98.5% for Process I and 96.7% for Process II, with corresponding yields of 78% and 65%, respectively. These integrated purification strategies allowed the successful recovery and purification of SA from the fermentation broth, providing valuable insights for efficient DSP of this important platform chemical [115].

*Strategy 5:* A two-step strategy was employed for the separation and purification of SA utilizing NF and RO membrane processes. Initially, the fermentation broth with 0.34 M succinate was diluted (2X) to a specific concentration of 0.175 M to enable the separation of succinate and acetate. In the first step, NF was employed in a diafiltration mode, where impurities including acetate, glucose, chloride ions, and phosphate ions were effectively removed, while succinate was retained with concentration of 0.16 M, and a significant increase in its purity from an initial 85% to an impressive 99.5%. The total yield of succinate remained high, surpassing 92%. Subsequently, in the second step, RO was used to concentrate the purified succinate solution, ultimately recovering the initial succinate concentration of 0.34 M from a diafiltrated solution of 0.16 M. With this integrated approach, the study achieved a remarkable separation and purification of SA, providing a high-purity product with a total yield exceeding 92% [116].

*Strategy 6*: In a recent study, pretreated spent sulphite liquor (SSL) derived from *Eucalyptus globulus* was used for *B. succiniciproducens* mediated SA production [117]. An optimal concentration of 12.5% activated carbon was required for the complete decolorization of fermentation broth containing 41.2 g/L SA. Furthermore, five different DSP strategies were evaluated for recovery and purification of SA: calcium precipitation, direct crystallization using acidification and cation-exchange resins, salting out, and reactive extraction. Among all the tested strategies, reactive extraction at pH-2 emerged as the best process with a solvent system comprising trioctylamine and *1-*hexanol, and back extraction of SA as sodium salt was 100%, using the pH swing method (with NaOH at pH-13). The entire process resulted in 73% SA recovery and SA crystals with a purity of 97.2%, when the sodium salt of SA was subjected to direct crystallization by cation-exchange resins [117].

*Strategy 7*: A multi-step DSP was employed for the separation and purification of SA from fermentation broth generated by *A. succinogenes* 130Z using industrial candy waste at 75L pilot-scale [118]. The initial step involved the clarification of the fermentation broth to remove biomass, color, and protein residues using activated carbon treatment, centrifugation, and ultrafiltration. This step resulted in a clear fermentation broth with minimal sugar and SA losses of about 9–10%. The subsequent purification steps were divided into two methods:

*Method 1:* Post-purification, the clear fermentation broth was treated with a cation-exchange resin (Amberlite IR 120 H), which converted succinate into SA. Approximately 78–83% of the SA in the broth was retained in the effluent after resin treatment, with about 17–22% SA loss during ion exchange. The effluent from this step was then subjected to NF to retain neutral molecules, such as residual sugars and proteins. NF enabled the retention of 94–96% of glucose and 100% of maltose, with only 8–10% SA rejection during NF. The NF was conducted after ion exchange, resulting in a treated broth with a pH of 2.1–2.6. The high molecular weight cutoff (MWCO) of the NF membrane relative to the molecular weight of the separated compounds allowed for the effective retention of sugars and neutral molecules. The concentrated retentate contained a high concentration of recovered sugars and nitrogen compounds. After the NF, the permeate was subjected to SA crystallization (4 ℃ and pH 2.0) and re-crystallization (4 ℃ and pH 2.0) followed by drying at 70 ℃, resulting in 84.9% recovery and 93.5% purity.

*Method 2:* Similar to Method 1, this method involved pre-purification. However, ion-exchange and NF steps were avoided. After the pre-purification, the broth was subjected to acidification (pH 2.0 using 95% sulfuric acid), followed by vacuum distillation to remove volatile acids (e.g., acetic acid and formic acid) and water while concentrating the solution. This step achieved a concentration of the solution to about 10–12% of its initial volume. The concentrated broth was then subjected to SA crystallization (4 ℃ and pH 2.0) and re-crystallization (4 ℃ and pH 2.0), followed by drying at 70 ℃. This method resulted in a SA recovery of 86.7%. However, the purity of the SA crystals obtained through this method was lower (26.2%) due to the presence of residual sugars and nitrogen compounds in the fermentation broth.

Overall, both methods achieved successful SA separation and purification, but Method 1 resulted in higher SA purity (93.5%) and Method 2 in higher SA recovery (86.7%). The results showed that SA recovery and purity can be optimized through different downstream strategies, and the choice between them may depend on specific requirements for SA quality and yield [118]. Table 4 lists some integrated techniques employed in improving the recovery yield and purity of SA from the fermented broth.

**Table 4:** Integrated techniques addressing the limitations for improved downstream processing

**Technique**	Recovery yield (%)	Purity (%)	Reference
Direct crystallization	70	90.0	[119]
Decolorization + Vacuum distillation + Crystallization	74.65	99.99	[120]
Vacuum distillation + Crystallization	28	45.0	[121]
Anion exchange + Crystallization	78	98.5	[115]
Microfiltration + Nanofiltration + Evaporation + Crystallization	86.5	99.2	[122]

## Catalytic upgrading of SA

Bio-based SA is a promising building block for a wide range of sustainable chemicals and a potential substitute of maleic acid in various applications [123]. γ-Butyrolactone (GBL), 1,4-butanediol (BDO), THF, N-methyl-2-pyrrolidone, 2-pyrrolidone, etc., are the notable industrially relevant SA derivatives, as shown in Fig. 1. The SA derivatives can be classified into four different categories based on their conversion chemistry: (i) hydrogenation products, (ii) esterification products, (iii) amination products, and (iv) others. This section presents chemo-catalytic upgrading of SA to these chemicals using various heterogeneous catalysts. The discussion encompasses the role of metals, supports, metal loadings, and reaction conditions on SA conversion and product yield/selectivity.

### Hydrogenation products

**Fig. 5:**
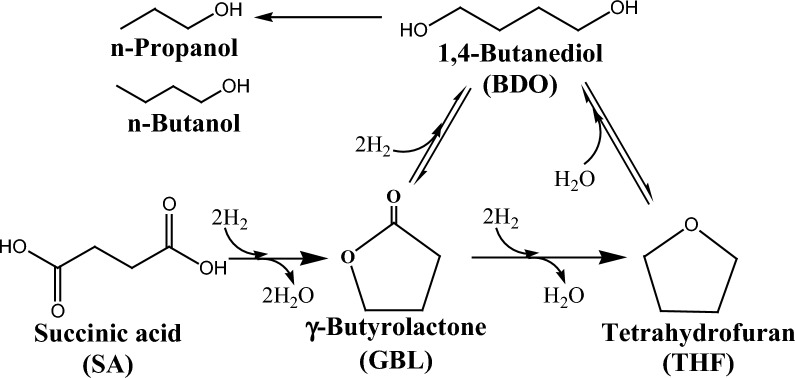
Reaction routes of SA hydrogenation to GBL, BDO, and THF

The hydrogenation of SA produces three important petrochemicals: BDO, THF, and GBL, as shown in Fig. 5. BDO is a versatile chemical with many commercial applications as an organic solvent and precursor for manufacturing fibers, adhesives, and polyurethanes [124]. It is also used as a starting material for producing various other chemicals, such as THF, GBL, and several polymers like polybutylene terephthalate and polybutylene succinate. At present, roughly 50% of BDO is consumed for THF production, whereas around 35% is used for producing various polymers, and the remaining amount is consumed in GBL production [125]. In the last few decades, there has been a growing interest in producing thermoplastics using BDO as a raw material [126]. The global BDO market was US $ 7.4  billion in 2023  and is anticipated to be US $ 14.11  billion by 2030 [127]. On the other hand, THF finds application as a solvent in the polyvinyl chloride manufacturing process and monomer for polytetramethylene glycol, which is an intermediate for polyurethanes and fibres manufacturing [128]. GBL is another important chemical and finds application as a solvent and raw material for manufacturing agrochemicals, pharmaceuticals, and rubber additives. These three chemicals (BDO, THF, and GBL) are currently produced from petroleum-derived maleic anhydride by hydrogenation reaction [124]. The bio-based SA opened another entry point for sustainable production of these chemicals through an integrated biorefinery approach [129].

The hydrogenation of SA is carried out using supported metal catalysts in the presence of hydrogen (Fig. 5) [126, 129, 130]. The process involves a series of consecutive hydrogenation reactions, in combination with dehydrocyclization and ring-opening reactions, forming GBL, BDO, and THF [131–133]. SA is first reduced to GBL, which is later hydrogenated to BDO and THF. Despande et al. investigated the hydrogenation of SA under mild reaction conditions and observed the formation of GBL, BDO, and THF as major products, with small quantities of *n-*propanol and *n-*butanol [131]. GBL, being an intermediate in the series of reaction networks, selectivity peaked at a certain reaction time and then decreased gradually with further progress of the reaction, with the concurrent increase in BDO selectivity [134, 135]. Operating conditions, such as SA concentration, temperature, and hydrogen pressure, are crucial for SA conversion and product selectivity. The SA conversion and the THF selectivity increased from 46.2% to 91.0% and 7.8% to 33.0%, respectively, whereas BDO selectivity decreased from 90.2% to 59.2% with increase in reaction temperature from 473 K to 513 K over Re/C-5 catalysts [129]. A similar trend was also observed for increasing hydrogen pressure, although the impact was much lower than temperature.

Several noble and transition metal-based catalysts are active for the aqueous phase hydrogenation of SA. Pd, Ru, and Rh metals were observed as active for SA hydrogenation (Table 5) [131–139]. The hydrogenation activity and selectivity to GBL, BDO, and THF differ based on the nature of support and metals, metal loadings, and operating conditions. For example, Pd/SBA-15 showed slightly higher hydrogenation activity compared to Pd/MCM-41 [136]. The higher catalytic activity of Pd/SBA-15 was due to the smaller Pd particles formed inside the sufficiently large SBA-15 pore channels. Au/TiO_2_ was also reported as very active with 97% SA conversion and selective for GBL [137]. Pt generally favors the hydrogen dissociation reaction with improvement in hydrogenation activity. The catalytic activity of Starbon-supported Pd, Pt, Rh, and Ru noble metals was thus tested for aqueous-phase hydrogenation of SA [138]. Ru and Pt catalysts showed higher catalytic activity than Pd and Rh due to smaller and evenly dispersed nanoparticles of Ru and Pt compared to Pd and Rh. While THF was dominant over the Ru catalyst, a high BDO yield was observed for the Pd, Pt, and Rh catalysts.

**Table 5:** Hydrogenation of SA over supported metal catalyst to produce BDO, GBL, and THF

Catalysts	Reaction conditions					X (%)	Selectivity (%)			Reference
	C (g/ml)	W (g/ml)	T (K)	P (MPa)	Reaction time (h)		BDO	GBL	THF	
Pd/SBA-15	0.11	0.06	523	10	8	65	36	39	25	[136]
Pd/Silica gel						57	25	27	48	
Pd/MCM-41						60	53	32	15	
Au/TiO_2_	0.3	0.004	523	11	10	97	-	97	-	[137]
5%Pt/Starbon	0.4	0.03	373	1	24	78	85	15	-	[138]
5%Pd/Starbon						75	70	30	-	
5%Rh/Starbon						60	90	10	-	
5%Ru/Starbon						90	10	30	60	
3 wt%Pd/C	0.2	0.005	473	5	10/50^**1**^	49.6	~ 41	~ 35	~ 6	[135]
3wt%Pd-1wt%FeOx/C						81.9	48	20.1	17	
3wt%Pd-5 wt%FeOx/C						87.7	70	~ 10	~ 10	
3wt%Pd-10 wt%FeOx/C						24.6	~ 22	~ 55	~ 3	
Re/C-5	0.11	0.011	473	8	10	46.2	0.8	90.2	7.8	[132]
			493			70.1	1.1	82.6	14.9	
			513			90.0	4.0	59.2	33.0	
			513 513	7		85.6	1.1	69.4	26.6	
				6		80.3	1.0	79.2	17.6	
0.6Re/MC	0.005	0.002	473	8	7	73.1	7.7	88.7	3.6	[134]
0.45Re–0.15Ru/MC						100	52.2	39.8	7.9	
0.3Re-0.3Ru/MC						100	71.2	18.1	10.7	
0.15Re–0.45Ru/MC						100	48.9	44.3	6.8	
0.6Ru/MC						45.2	1.8	97.5	0.8	
Co/C	0.22	0.06	523	10.5	5.5	20	–	- ~ 55^Y^	~ 3.3^Y^-	[131]
Ru/C					7		–	~ 78^Y^-	~ 11^Y^ -	
1%Ru–Co/C					2		~ 33^Y^ -	~ 61^Y^ -	~ 6^Y^ -	
Ni/SiO_2_	0.01	0.001	473	6	6	45	-3^Y^	4^Y^	–	[139]
Co/SiO_2_						36	- ~ 11^Y^	~ 3^Y^ -	–	
Co/SiO_2_–Al_2_O_3_						28	~ 2^Y^	~ 3^Y^	–	
Co/Al_2_O_3_						38	–	–	–	

X: Conversion of SA, Y: Yield, C: SA concentration, W: Amount of catalyst. ^**1**^Conversion at 10 h and selectivity at 50 h

Hydrogenation of SA was further studied over FeO_*x*_-promoted 3 wt% Pd/C catalysts with different Fe content to evaluate its role as a promoter [135]. The conversion of SA was only 49.6% for unpromoted Pd/C catalyst and increased to 81.9% and 87.7% by adding 1 wt% and 5 wt% FeO_*x*_, respectively. However, the conversion of SA dropped to merely 24.6% for 10 wt% FeO_x_. The addition of FeO_x_ in Pd/C simultaneously promotes total acidity with enhanced dehydration activity and encapsulation of Pd particles with reduced metal dispersion, bigger particle size, and reduced hydrogenation activity. The proper balance of acidity and metal dispersion is thus critical for high catalytic activity and selectivity of the desired product. The catalytic activity of FeO_*x*_-promoted Pd/C catalysts was thus enhanced with increased addition of FeO_x_ up to 5 wt% due to an increase in total acidity and dropped drastically at 10 wt% FeO_x_ content owing to poor metal dispersion. Besides, BDO selectivity was improved with increasing FeO_x_ loading up to 5 wt% with a simultaneous decline in GBL selectivity due to synergistic interaction between Fe and Pd species and decreased drastically at 10 wt% FeO_x_ loading.

A series of mesoporous carbon (MC)-supported Re–Ru bimetal catalysts were evaluated to understand the effect of the Re/Ru metal ratio on catalytic activity [134]. Re and Ru monometal catalysts showed merely 73.1% and 45.2% SA conversion, while almost complete SA conversion was observed over Re–Ru bimetal catalysts. The improved catalytic activity of bimetal catalysts was due to the synergistic Re–Ru interaction and formation of a solid solution of Re–Ru during the reduction. Besides, the ease of reduction, metal dispersion, and oxidation state of metals were greatly affected by the Re/Ru mole ratio. 0.3Re–0.3Ru/MC displayed the maximum BDO turnover frequency due to the highest amount of weak hydrogen-binding sites. 0.3Re–0.3Ru/MC also demonstrated good stability and reusability.

The catalytic activity of carbon-supported Co, Ru, and bimetal Ru–Co catalysts was investigated [131]. Ru, Co, and RuCo catalysts exhibited complete conversion of SA in 7 h, 5.5 h, and 2 h, respectively. The enhanced hydrogenation rate for the bimetal catalyst was due to the synergistic effect. BDO formation was found to be independent of Ru content in RuCo bimetal catalysts. The liquid-phase SA hydrogenation was further studied over SiO_2_, Al_2_O_3_, and SiO_2_–Al_2_O_3_ supported Ni and Co catalysts [139]. The highest BDO selectivity at 20% SA conversion was observed over Co/SiO_2_ compared to Ni/SiO_2_, Co/Al_2_O_3_, and Co/SiO_2_–Al_2_O_3_.

### Esterification products

**Fig. 6:**
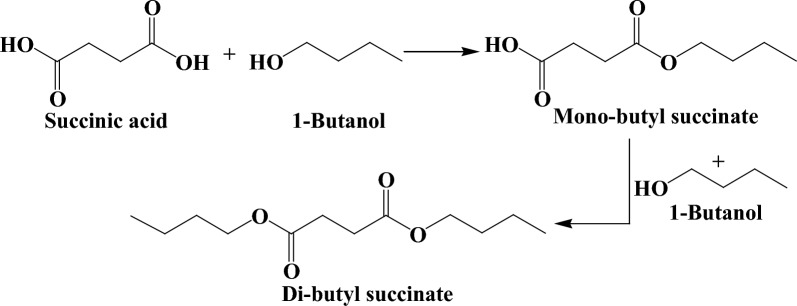
Esterification reaction of SA with *1-*butanol

The esters of SA, i.e., succinates, such as mono and di-butyl succinate, have numerous industrial applications, including plasticizers, scents in cosmetics and food products, perfumery, diluents in paints and coatings, drug intermediate, and dyes [140]. They are also used as a green solvent. The succinates, especially dibutyl succinate, are promising candidates for fuel additives due to their low water miscibility [141, 142]. The acid-catalyzed esterification reaction involves the formation of carbocation by protonation of the carboxylic group, followed by nucleophilic attack by the alcohol group, forming the corresponding monoester (Fig. 6) [143]. The same reaction mechanism is repeated for esterification of the second carboxylic group present in the monoester. The esterification is an autocatalytic reaction, but the reaction rate is very slow, taking 48 h for its completion. The water inhibits the esterification reaction due to the promotion of reverse reaction, i.e., hydrolysis and competitive protonation by water with alcohol [144]. Several heterogeneous acid catalysts were explored for the esterification of SA. The degree of esterification and selectivity to mono and di-succinate strongly depends on the types of catalysts and their acidity and operating conditions. For example, the catalytic activity of montmorillonite clay (mont) exchanged metal ions (M^n+^: Al^+3^, Fe^+3^, Cr^+3^, Ni^+2^, Zn^+2^, Mn^+2^, and Na^+^) was tested for esterification of SA with various alcohols including *1-*butanol and phenol (Table 6) [143]. The catalytic activity of these catalysts was independent of surface area and acidity but correlated linearly with the charge/radius ratio of M^n+^ ion, with the highest being for Al^+3^ [143]. The Al^+3^-mont catalyst thus exhibited the highest esterification activity with 94% yield of diester. The effect of solvent for esterification reaction was further studied using Al^+3^-mont catalyst. While the polar nature of dioxane inhibits ester formation, 94% and 86% diester yield was observed for toluene and xylene, respectively. However, only 11% diester yield was observed for benzene due to its low boiling point.

**Table 6:** Solid-acid catalysts for the esterification of SA

Catalyst	Reaction conditions				X (%)	Yield (%)		Reference
	T (K)	Solvent	Reaction time (h)	Acid/alcohol		Monoester	Diester	
mont clay	Reflux	Toluene	8	1:3	100	–	0	[143]
Al^+3^-mont						–	94	
Fe^+3^ -mont						–	81	
Cr^+3^_-_mont						–	79	
Ni^+2^-mont						–	46	
Zn^+2^-mont						–	36	
Mn^+2^-mont						–	18	
Na^+^-mont						–	0	
Al^+3^-mont	Reflux	Toluene				–	94	
		Xylene				–	86	
		Benzene				–	11	
		Dioxane				–	0	
No catalyst	353	Ethanol	4	1:3	24	80	10	[144]
H_2_SO_4_					> 95	33	67	
Starbon-400-HSO_3_					90	20	80	
DARCO–HSO_3_					60	78	18	
NORIT–HSO_3_^**a**^					70	68	29	
Carbon-P-250					52	79	21	
(A)Carbon-P-250					78	68	23	
Starbon-400-HSO_3_			1		52	71	22	
			8		> 95	< 5	> 95	
MCM-22	363	–	10	1:3	64	64	36	[145]
SiW12					100	02	98	
SiW12/ MCM-22					97	41	59	

^**a**^ 8 h reaction time

The catalytic activity of sulfonated mesoporous Starbons was studied for SA esterification with aqueous ethanol [144]. This catalyst showed greater than five times catalytic activity and quantitative yield of diester within 5 h compared to the other microporous carbon-based solid-acid catalysts with similar acidity. The promising catalytic activity of Starbon-400-HSO_3_ was due to enhanced molecular diffusion of reactants/products to/from mesopores and an ideal combination of hydrophilic/hydrophobic properties. More than 95% SA conversion with > 95% diester selectivity was achieved at 8 h reaction time using Starbon-400-HSO_3_ catalyst.

The catalytic activity of MCM-22, SiW_12_, and SiW_12_-MCM-22 was evaluated for the esterification of SA [145]. About 64% SA conversion was reported over MCM-22 with 36% diester selectivity compared to complete SA conversion with 98% diester selectivity over SiW_12_ and 97% SA conversion with 59% diester selectivity over SiW_12_-MCM-22. The higher catalytic activity and diester selectivity over SiW_12_-MCM-22 than MCM-22 was due to the higher SiW_12_-MCM-22 acidity.

### Amination products

2-Pyrrolidone (2P) has numerous applications in manufacturing agrochemicals, medicines, and pharmaceuticals and is a useful chemical in producing nylon-4 types of polymers [129]. Bio-based 2P and N-methyl-2-pyrrolidone (NMP) are also recognized as green non-volatile solvents [146]. The high boiling point of NMP makes it a suitable replacement for chlorinated solvents with lower volatile organic compound emissions. More specifically, NMP is used as a solvent for high melting point polymers, such as polyurethanes, polyacrylonitriles, and heterocyclic [147]. NMP also finds application in solvent extraction of acetylene and butadiene. BASF, Lyondell-Basel, ISP-Ashland, and Mitsubishi are major players around the world in manufacturing 2P and NMP by petrochemical routes. 2P and NMP are generally produced by the reduction of SA using amine, ammonium, ammonia, and optionally alcohol over catalysts, known as reductive amination [129]. Previously, 2P was synthesized from propylene oxide through BDO.

**Fig. 7:**
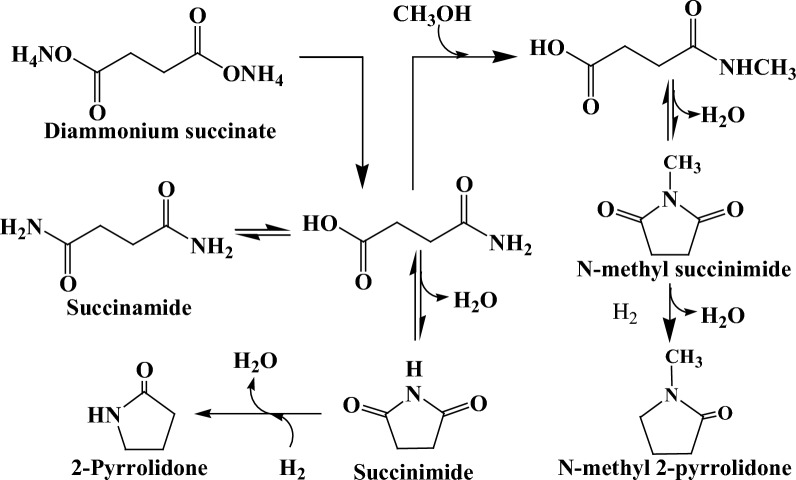
Reaction routes for synthesis of 2-pyrrolidone and N-methyl-2-pyrrolidone from SA

A few patents are available on the synthesis of 2P from SA without GBL intermediate, with limited information on the reaction mechanism. A possible reaction mechanism for producing 2P and NMP from diammonium succinate is shown in Fig. 7, as reported by Werpy et al. [148]. The synthesis involves the reaction of succinate with ammonia, with the formation of diammonium succinate. The diammonium succinate then undergoes a reversible dehydration reaction to produce succinimide. Succinimide is then hydrogenated to produce 2P with simultaneous removal of water. On the other hand, methanol is added to synthesize NMP from diammonium succinate.

The conversion of SA to 2P and NMP depends on the selection of metal catalyst, reaction condition, and ratio of reactants. Cobalt, nickel, ruthenium, palladium, Raney cobalt, and Raney nickel are the most active catalysts for selective production of 2P from SA (Table 7) [148–153]. Al_2_O_3_-supported Ru catalyst showed a higher yield of 2P compared to Raney cobalt and Raney nickel [151]. The high catalytic activity and 2P selectivity of the Ru catalyst were due to its ability to withstand catalyst poison. On the contrary, Ru–Re bimetal catalysts showed higher selectivity towards NMP [148]. The suitable reaction parameters are essential to obtain the desired 2P or NMP. Ethanol with amines gives NMP as the desired product during reductive amination of SA [129, 148]. The temperature above 563 K improves the yield of 2P [152].

**Table 7:** Catalysts for synthesis of 2P and NMP from SA

Catalyst	Reaction condition			X (%)	2P Yield (%)	Reference
	T (K)	P (MPa)	Reactant ratio			
Raney cobalt	511–531	9–11	3.1:1	–	30–69	[149]
Co	573	25	2:1	–	79	[150]
5%Ru/Al_2_O_3_	525	11.7	1.5:1	–	92	[151]
5%Pd/Al_2_O_3_	543	17.2	1.1:1	–	~ 62	[152]
RuFeNiOx	523	6.9	2:1	–	77	[153]
2.5%Rh–2.5%Re on C	538	13	2:1	~ 91	90^ M^	[148]

M: Maximum yield of NMP + 2P

### 1,4-Diaminobutane and succinonitrile

Succinonitrile is considered the precursor of 1,4-diaminobutane for producing polyamides. It is commercially produced using acrylonitrile and hydrogen cyanide [154]. Biomass-derived succinonitrile can be a potential alternative for sustainable production of polyamides, such as nylon. Besides, the synthesis of polyesters and poly(ester amide)s requires 1,4-diaminobutane. Succinonitrile can be produced from biomass-derived SA during fermentation [130]. The succinonitrile is synthesized via the reaction of ammonia with SA using Si_3_PO_4_ catalyst at 693 K [155]. Ammonium carbonate is also used as a reagent for the synthesis of nitriles from carboxylic acid. For example, benzoic acid reacts with ammonium carbonate at given reaction conditions and forms benzo nitrile with 90% yield [156]. Lammens et al. [157] also reported the synthesis of bio-based succinonitrile from glutamic acid and glutamine. They reported 100% yield of succinonitrile at 62% conversion of succinimide-derived 3-cyanopropanoic acid using Pd(II) catalyst with the reaction of acetonitrile. They also reported the formation of SA from glutamine via oxidative decarboxylation.

## Commercial players in bio-SA production

The commercial production of bio-based SA has already been proven by several companies, as shown in Table 8 [158–163]. The first commercial plant for bio-based SA was opened in 2012 by Reverdia, a joint venture (JV) of DSM and Rouquette. The former company developed Biosuccinium^®^ technology, which was licensed to Rouquette. The proprietary technology involved a recombinant *Saccharomyces*-based low pH fermentation process, in which the yeast produced SA from sustainable biomass-derived carbon sources or starchy feedstocks [161, 164, 165]. Later, this JV was dissolved in April 2019, and the exclusive rights were owned by DSM [158]. In August 2022, Technip Energies bought the Biosuccinium® technology from DSM, which included several yeast strains as well as patent portfolios associated with SA production. The company envisages using bio-based SA to synthesize polybutylene succinate or PBS biopolymer [166]. Thereafter, Myriant, a US-based company, the pioneer in bio-based SA production using recombinant *E. coli* strain, opened its first commercial plant in 2013. Their Louisiana-based plant was funded by the Department of Energy (DoE) and used grain sorghum as the starting feedstock [167]. However, the company could not match the techno-economics of the finished product with fossil-based routes and stalled its Louisiana plant operation in 2016. In 2021, Stepan bought this fermentation facility to start commercial rhamnolipid production. Myriant opened its second plant in Leuna, Germany, which was managed by their collaborator and engineering partner, ThyssenKrupp Uhde [160]. However, the status of this plant is unclear. In 2014, Succinity, the third industrial entity, opened a commercial plant in Spain, harnessing the potential of * B. succiniproducens*. It was a JV between Germany-based BASF and Dutch company Corbion Purac [161]. This venture broke in 2019 due to the high cost of bio-based SA compared to the petrochemical route (reduced crude oil prices in 2018) and its dwindling commercial prospects [162]. However, as per the recent report [16], BASF is a leader in bio-based SA, suggesting that the plant is still operational. BioAmber, initially a JV between New York-based Diversified Natural Products (DNP) and France-based ARD, was another company that began largest commercial bio-SA production in 2015, with proprietary acid-tolerant yeast, exclusively licensed by Cargill. Jointly with Mitsui Japan, they chose Canada’s Sarnia for its commercial operation, but unfortunately they had to file for bankruptcy in 2018. The failure of BioAmber’s Sarnia plant was a culmination of several factors; for instance, the actual cost of the SA was ten times higher than the predicted cost, the market of SA was overestimated, and falling prices of petrochemical-based SA, the over-reliance on licensed technologies rather than expanding their patent portfolio and not running the plant to its full capacity, as reviewed by Li and Mupondwa [168].

**Table 8:** Commercial players involved in bio-SA production

Manufacturer	Organism used	Production capacity	Start year	Location	Details and present status	Reference
Reverdia, a JV of DSM and Rouquette	Recombinant *S. cerevisiae*	10,000 MT/year	2012	Cassano, Spinola, Italy	• In 2019, this JV dissolved and exclusive rights went to DSM • In 2022, the technology was purchased from DSM by Technip Energies	[158]
Myriant	Recombinant * E. coli*	14,000 MT/year	2013	Lake Providence, Louisiana, USA	• In 2016, SA production stopped • In 2021, Stepan Company acquired this plant for rhamnolipid manufacturing	[159]
ThyssenKrupp Uhde		500 MT/year	2013	Leuna, Germany	• Myriant was their technological partner • Current status: unknown	[160]
Succinity, JV of Corbion Purac and BASF	Recombinant* B. succiniciproducens*	10,000 MT/year	2014	Montmeló, Spain	• The JV liquidated in 2019 and presently only BASF is manufacturing bio-based SA	[161] [162]
BioAmber in collaboration with Mitsui Chemicals	Recombinant *Candida krusei*	30,000–50,000 MT/year	2015	Sarnia, Canada	• In 2018, BioAmber declared bankruptcy • LCY Biosciences Inc, a Taiwan-based company acquired this plant in 2018 and ramped up its production capacity from 8000 to 18,000 metric tons (MT) in 2021	[158, 163]

## Challenges associated with biological SA production

Enormous progress has been made in the biological production of SA, and some of the challenges have been alleviated, but it is insufficient for reliable bio-SA production. The bio-based production of SA is promising commercially as well to a certain degree, but more work is needed to compete with fossil-based production. In recent times, SA manufacturing via fermentation has been declining due to the higher price tag of bio-based SA (US $ 2.86–3.00/kg) in comparison to fossil-based SA (US $ 2.40–2.60/kg) [6]. To this end, various challenges need to be overcome.

### Low price feedstocks

The feedstock cost is one of the major factors deciding the industrial viability of a fermentation-based product. Therefore, adopting low-priced feedstocks becomes critical for envisaging a profitable bioprocess [169]. The substrate cost for fermentative SA could be reduced to US $ 0.53–0.75 with renewable carbohydrate feedstocks and contribute to a process with high commercial competitiveness [6]. As mentioned above, SA can be obtained from several carbon sources, including corn cob, corn fiber, sugarcane bagasse, molasses, whey, crude glycerol, etc. [4, 6]. The perusal of the literature shows that there is a large variety of cheap and abundant crude renewable sources, such as agricultural residues, forest biomass, and industrial and unavoidable waste streams from the supply chain. However, developing conversion technologies to obtain cheap fermentable carbon devoid of inhibitors from these sources is a big challenge. Thus, the economical production of clean fermentable sugars from non-edible biomass needs to be advanced. Furthermore, different feedstocks are rich in different carbon sources, and sometimes a feedstock contains multiple carbon sources, which further complicates the situation, as assimilatory, transport, and regulatory mechanisms remain poorly understood for non-conventional carbon sources and require intensive research work in this direction [6]. In the case of a mixture of sugars with glucose, carbon catabolite repression suppresses the metabolism of other sugars [11]. The production medium for SA also requires a high concentration of expensive complex nitrogen sources, such as yeast extract, YNB, etc., which can be replaced with cheap nitrogen sources, including corn steep liquor and spent brewer’s yeast hydrolysate, or by a bioresource providing both carbon and nitrogen, e.g., waste bread, potatoes, and wheat milling byproducts [4, 6, 170, 171].

### Tools for designing robust hyper-accumulating cell factories

Currently, there are several bacterial and yeast strains accumulating SA > 100 g/L. However, yield and volumetric productivity need to be significantly improved to make SA cost-competitive with fossil-based production. As shown in Tables 1 and 2, the product yield is substantially lower than the theoretical yield, and productivity is much lower than the industrial requirement (> 3.0 g/L. h) in most cases. The low yield implies consuming a large amount of substrate with high operational costs, while lower productivity signifies gigantic fermenters with excessive capital investment. Bacterial strains are sensitive to pH fluctuations and require pH control throughout the process, whereas yeast cell factories are tolerant to low pH and can continuously accumulate SA without controlling pH [6, 13]. However, the downside of yeast strains is that they require longer fermentation time, leading to low productivity. Furthermore, unlike bacterial strains, yeast cell factories employ an oxidative TCA cycle with low theoretical yield. All these factors make them uncompetitive to bacterial hosts and limit their industrial applications [6]. Though native bacterial SA producers are quite promising, research on genetic manipulation of these strains has not been done so far. For example, *A. succinogenes,* well-studied bacteria, lacks effective genetic modification tools to carry out extensive metabolic engineering for further improvement in SA fermentation [4, 5, 9]. On the contrary, efficient genetic tools are available for yeast strains, such as *S. cerevisiae* and *Y. lipolytica* [97, 100]. These strains have been genetically manipulated to obtain SA in large amounts. End-product toxicity is well known, and organic acids are more toxic than alcohols. Organic acids are known to reduce the cytoplasmic pH, which causes deleterious impacts on cellular machinery [172]. The work in this direction relies on evolutionary engineering techniques and random mutagenesis. Attention should be paid to decoding the underlying mechanism for the rational designing of evolved strains and key enzymes. Therefore, the construction of a robust strain for overproduction of SA with high yield and productivity at low pH (< 4.0), along with high tolerance against SA, is essential for industrial-scale production economically. This is the greatest challenge that we have at current times. The current metabolic engineering approaches are quite straightforward, including overexpression of pathway genes and deletion of byproducts. Despite the overexpression of pathway enzymes and elimination of pathways leading to byproducts, the carbon loss could not be abolished entirely to improve SA production. However, the regulatory network controlling the biochemical pathways is quite complex, and expected results that match industrial requirements are difficult to achieve with frequently used simple genetic engineering methods. For example, global regulators can be good targets to overcome the limitations associated with classical metabolic engineering approaches [173]. Furthermore, strains should be designed to expand their ability to assimilate non-conventional carbon sources, which will help in harnessing the full potential of renewable sources [174]. The advancement in systems and synthetic biology, metabolic and evolutionary engineering, along with the availability of powerful tools, gives a strong hope that these modern techniques, individually or in combination, can lead to the designing of robust strains accumulating SA with high TYP metrics, leading to efficient and low-cost production.

### Redox balance

Among the three routes for the biological production of SA, the most promising is rTCA in terms of product yield and carbon capture. SA is a reduced fermentation product, consuming four electrons to form one molecule [5]. The pathway requires two moles of reducing equivalents to generate one mole of SA. Often, biosynthesis of SA via this pathway encounters NADH deficiency, as glucose catabolism to pyruvate generates only half of the requirement. Thus, a sufficient amount of NADH is a prerequisite and crucial for achieving a high-level SA production [173]. This NADH deficiency has a negative impact on the biosynthesis of SA and restricts the yield of glucose to 1.0 mol/mol. To overcome this, certain NADH-generating enzymes can be introduced, such as formate dehydrogenase, transhydrogenase, etc., to enhance the NADH pool [175]. Additionally, supplying reduced carbon sources or carbon sources with higher oxidation states and higher NADH yield, such as glycerol (2 NADH/mol), sorbitol (3 NADH/mol), and/or addition of extra electron donors, such as hydrogen, could be another way to alleviate this problem [5, 176]. At the same time, NADH-consuming pathways, especially leading to byproduct formation, such as lactic acid, ethanol, 2,3-butanediol, etc., should be inactivated. The presence of exogenous electron acceptors, including molecular oxygen, should be optimized or minimized to the extent that the NADH/NAD^+^ ratio becomes favorable for SA accumulation. Besides consuming NADH, these byproducts consume a substantial amount of substrate carbon and, thereby, significantly diminish the SA yield. Regulation of redox potential can also have a positive influence on SA yield and productivity with low byproduct formation [5, 169].

### CO_2_ supply

One of the fascinating features of rTCA is that it requires CO_2_ as a co-substrate for SA production. The concentration of dissolved CO_2_ regulates the activity of carboxylating enzymes and is an influencing factor in diverting carbon from the main substrate towards SA and deciding the ratio of SA to byproducts [4]. It has been found that less SA is synthesized under limiting CO_2_ levels, and enhanced CO_2_ concentration stimulates SA production and reduces byproduct formation. The available forms of CO_2_ in fermentation broth are HCO_3_^−^, CO_3_^2−^, and CO_2_, influenced by medium composition, pH, temperature, agitation speed, flow rate, and partial pressure [9, 177]. The affinity of carboxylating enzymes for CO_2_ fixation is low, indicating the need for high partial pressure to divert C3 metabolites toward the SA pathway [178]. Since the solubility of CO_2_ is poor at atmospheric pressure, the culture medium is supplemented with carbonate and bicarbonate salts as indirect sources of CO_2_ to improve the dissolved levels of the gas [179]. The transport of HCO_3_^−^ through the cell membrane via passive diffusion is very slow, and one of the approaches to troubleshoot this problem could be to make use of a bicarbonate transporter (SbtA and BicA) for enhancing levels of HCO_3_^−^ [180, 181]. However, PEPCK prefers CO_2_ with higher catalytic velocity (7.6-fold) over HCO_3_^−^, and one of the ways to promote the intracellular conversion is the introduction of carbonic anhydrase, which efficiently converts HCO_3_^−^ into CO_2_ [181, 182]. In the current time, due to lots of interest in carbon sequestration, storage, and utilization, the availability of CO_2_ is huge, with a market price of US $ 60–450 per MT. In fact, the biogenic CO_2_ stemming from fermentative processes, such as ethanol, 2,3-butanediol, and anaerobic digestion, could be integrated with SA bioproduction. Since transportation contributes significantly to the total cost, the ideal situation would be capture, storage, and production sites next to each other [183, 184]. Furthermore, different microbes have different tolerance levels of CO_2_ and should be individually optimized for each microorganism under specific culture conditions [9].

### Economical downstream processing (DSP)

For bio-SA or any other bio-based product, the economic viability is deeply intertwined with the efficiency and cost-effectiveness of unit procedures employed in the DSP. High recovery and high purity of SA from the fermentation broth using advanced and cost-effective approaches is mandatory for the commercial success of the bioprocess. Economical DSP is pivotal in shaping the overall production cost, minimum selling price, or the market price of bio-SA. A recent exclusive review on bio-based SA clearly shows that the cost contribution of DSP ranges between 60–80% [185]. Besides high purity and yield of SA, capital expenditure associated with DSP, and number of unit operations for the said module, DSP should minimize resource (material/chemical/energy) consumption and reduce waste generation, thereby lowering the environmental footprint. The fermentation broth, from which SA is extracted, often contains impurities and byproducts. Separating and purifying SA from this complex mixture poses a significant challenge, as conventional methods may be less effective or require additional material and technological advancements. Besides, these processing steps can be energy-intensive. High energy consumption contributes to operational costs and contradicts the sustainability goals of bio-based production. Finding energy-efficient alternatives becomes essential to enhance the economic viability of bio-SA. Finally, the process should be commercially viable in higher volumes, maintaining efficiency and cost-effectiveness. Large-scale DSP requires innovative solutions to overcome issues related to equipment design, process integration, and economies of scale.

Potential solutions include the implementation of combined advanced separation technologies, such as membrane filtration, chromatography, and ion exchange, which improve the recovery and purification efficiency of DSP. These methods should offer higher selectivity and specificity, enabling more precise separation of SA from impurities. Furthermore, continuous efforts in process optimization can streamline DSP, reducing the number of steps and overall complexity. This can lead to significant cost savings and improved efficiency, making bio-SA more economically attractive. Additionally, integrating renewable energy sources, such as solar or biomass-derived energy, into DSP can minimize the environmental impact and operational costs. With these advancements, bio-SA can emerge as a leading bio-based chemical, offering a green alternative to traditional petrochemical routes and contributing to a more sustainable and resilient industrial landscape.

### Robust catalyst

Integrated fermentation and chemo-catalysis is a novel approach to obtain a range of chemicals from SA. However, unlike the petrochemical production of SA, fermentative production involves challenges in producing contamination-free pure SA. These contaminants may originate from micro and macro nutrients added in the fermentation and complex steps involved during the DSP of SA from aqueous fermentation broth. Besides, the separation and purification of SA from fermentation broth is energy-intensive. Therefore, directly upgrading SA fermentation broth is another approach to save energy consumption for SA separation. However, till now, analytical grade SA has been used for its conversion to various chemicals to evaluate the performance of the catalysts. Given the above facts, chemo-catalytic SA upgrading should also be studied using fermentative SA to design and judge the robustness of the catalyst system with entirely different types of contaminants. Studies should be done on different purification approaches, especially when SA fermentation broth is used as a starting material. Besides, studies should also focus on bench-scale or pilot-scale for tuning the process conditions.

### Sustainability assessment

Sustainable SA production from biomass and chemo-catalytic upgrading to valuable chemicals is essential for making the SA platform successful in biorefinery and an alternative to petrochemicals. It depends on many factors, including the availability of low-cost biomass feedstock, economic competitiveness with petrochemical routes, and environmental benefits/impacts associated with these processes. In this direction, extensive studies have been reported on techno-economic and life cycle analysis for fermentative production of SA from various biomasses and wastes (Table 9) [186–191]. SA production costs or selling prices vary depending on the type of feedstock and plant capacity. However, bio-based SA is economically feasible for centralized biorefineries with large plant capacity. The GHG emission potential is significantly lower compared to the petrochemical route. However, techno-economic and life cycle analysis for bio-based SA-derived valuable products is scarce in the literature. For example, cradle-to-grave life cycle analysis showed that the GHG emission for bio-based BDO production via SA hydrogenation was 52% lower than the fossil-based route [191]. Recently, Haus et al. reported the techno-economic and life cycle analysis for bio-based N-vinyl-2-pyrrolidone production by amidation–hydrogenation of SA with ethanolamine using different catalysts to N-(2-hydroxyethyl)-2-pyrrolidone, followed by its dehydration [190]. The results were further compared with fossil-based N-vinyl-2-pyrrolidone production from GBL. The manufacturing cost of bio-based N-vinyl-2-pyrrolidone (US $ 4.6–6.3/kg) was competitive with the fossil-based route (US $ 5.4/kg), with a significant reduction in global warming potential. Further sustainability assessment studies are needed to demonstrate the challenges and feasibility of SA derivatives.

**Table 9:** Economics and environmental impacts of bio-based succinic acid and its downstream products

Product	Feedstock	Plant capacity (MT/day)	Production cost (Selling price) (US $/kg)		Global warming impact (kg_CO2_ eq./kg)		Reference
			Bio-based	Fossil-based	Bio-based	Fossil-based	
Succinic acid	Sugarcane bagasse	96, biomass (dry)	1.61 (2.37)	–	1.39	–	[186]
	Pulp log trees	2,000, biomass (dry)	0.4 (0.93)	–	–	–	[187]
	Wine waste	82, SA	1.23–2.76 (4.42)	(2.94)	1.47	–	[188]
	Corn/sugarcane	–	–	–	0.88–1.70 /0.88–1.94	8.82	[14]
	Sugarcane bagasse	32.1, biomass (dry)	2.32	–			
	Bread waste	–	–	–	0.87–1.3	1.94	[189]
N-Vinyl-2-pyrrolidone	SA	–	4.6–6.3	5.4	3.5–5.7	7.6	[190]
1,4-Butanediol	SA	–	–	–	1.9	–	[191]

## Future perspectives and conclusions

In recent years, noticeable climate change has been witnessed worldwide due to the over-exploration of petroleum. This inevitable concern demands enforcing and shifting toward green and hybrid technologies for the sustainability of the chemical sector, including SA and its derivatives [16]. The SA is an acclaimed bio-privileged platform chemical. SA production is thus expected to pace up in the current decarbonization era, as one of the biosynthetic pathways uses reverse or rTCA cycle, involving CO_2_ fixation. Sinopec China has recently claimed a successful development of biorefinery technology for manufacturing SA using CO_2_ as a raw material [192]. However, bio-based SA is costlier than petroleum-based SA. Therefore, the market acceptability of bio-based SA demands a government policy framework to facilitate innovative technological developments to achieve environmental sustainability and foster the bio-based economy. This review provides a detailed discussion of the challenges and potential solutions to circumvent the hurdles associated with bio-SA production.

Substantial research progress has been made in obtaining robust hyper-SA-producing strains and DSP of SA from fermentation broth. However, the promising SA separation methods are mostly demonstrated on a laboratory scale. These technologies must be tested at a pilot scale to validate their commercial suitability. It is also equally essential to choose the right system boundaries to conduct life cycle assessment and techno-economic analysis for accurate predictions, as these tools are highly dependent on the date fed, and standard ISO guidelines should be adopted. The lesson must be learned from the failure of BioAmber’s Sarnia plant, which was primarly due to the overshooting of SA production cost by ten times the estimated manufacturing cost. Therefore, companies must evaluate the risks and *ex-ante* techno-economic analysis of the entire integrated process at the pilot-scale or pre-commercial scale before relaunching bio-SA commercially [168]. It is also equally crucial to meticulously revisit the other causes of the failure of commercial and pre-commercial SA plants despite the trailblazing launch of the bio-SA way back in 2012.

Despite initial setbacks and even bankruptcies, the resurgence of this nearly-dead industry has already begun. The business entities have started addressing the technical, non-technical, and administrative glitches with due diligence. They are also conscientiously assessing the uncertainties and risks involved in running this highly volatile business and have evolved a mitigation strategy to make bio-SA cost-competitive with the petrochemical counterpart. For instance, Myriant, now GC Innovation America, has re-entered the SA business, where the targeted product is PBS [193, 194]. Likewise, the Sarnia plant of BioAmber was bought by Taiwan-based LYC Company. Later, they ramped up the plant operations from 8000 MT/year to 18,000 MT/year in 2021 and are expected to operate the plant at full capacity in the near future [195]. BioAmber also shared its technical know-how with Taiwan-based PTT–MCC, a JV of Mitsubishi and PTT–PLC. They are using BioAmber’s SA fermentation technology for PBS production. Presently, their 20,000 MT/year polybutylene succinate plant in Rayong, Thailand, is using BioAmber’s fermentation technology to produce bio-based SA, which is the starting feedstock to produce this biopolymer. The bio-SA has been anticipated to be embarked upon considering these industrial advancements.

## Concluding remarks

The present review comprehensively presents all the aspects of microbial metabolism and bioprocesses involved in bio-based SA production, including commercial breakthroughs. Its pivotal role as a chemical building block and valorized products obtained using the chemo-catalytic route, the challenges associated with its production and future prospects are dealt in detail. Under the prevailing scenario, in order to foster the circular economy, resource recovery, maximizing use of renewable carbonaceous feedstocks, waste minimization, and energy integration are highly essential, as they also strengthen environmental sustainability. Authors are optimistic that the cumulative efforts by industries and government to revive the bio-SA technology, unprecedented climate change, increasing global awareness towards switching from fossil-based SA to bio-SA, and strong and positive market sentiments will surely revitalize and resurrect commercial production of bio-based SA.

## Data Availability

All the data has been submitted and there is no other available data and materials.
